# Molecular Conformation as a Determinant of Self-Assembly in Sequence-Isomeric Tripeptides

**DOI:** 10.1021/acs.jpcb.6c03754

**Published:** 2026-10-12

**Authors:** Zhuojun Li, Rein V. Ulijn, Hamish W. A. Swanson

**Affiliations:** † 2009 Advanced Science Research Center (ASRC) at the Graduate Center of the City University of New York, 85 St Nicholas Terrace, New York, New York 10031, United States; ‡ Department of Chemistry, Hunter College, City University of New York, 695 Park Avenue, New York, New York 10065, United States; § Ph.D. Programs in Chemistry and Biochemistry, The Graduate Center of the City University of New York, New York, New York 10016, United States

## Abstract

In this work, we evaluate whether computed conformational ensembles of seven tripeptides—the six C-terminal amidated D/Y/F sequence isomers and the closely related sequence FFD—are informative of their self-assembly propensity. Using all-atom molecular dynamics (AA-MD) simulations and intramolecular side-chain dihedral angles as a collective variable, we calculate conformational free-energy landscapes (FELs) for these sequences. We then introduce a novel method to numerically characterize the computed FELs and find a correlation between thermal conformational accessibility and a sequence’s tendency to self-assemble or remain in solution. In addition, we evaluate and rationalize the impact of water model selection on conformational ensembles in terms of peptide-water interaction strengths, and also investigate the influence of ionization state on conformation. The presented methodology implicitly accounts for amino acid sequence context, which is a major determinant of divergent properties for sequence isomers. It also has a low associated computational cost, making use of single-molecule simulations to predict supramolecular assembly outcomes. These features are an important advancement toward a general scheme for the theory-informed design of peptide materials.

## Graphical abstract

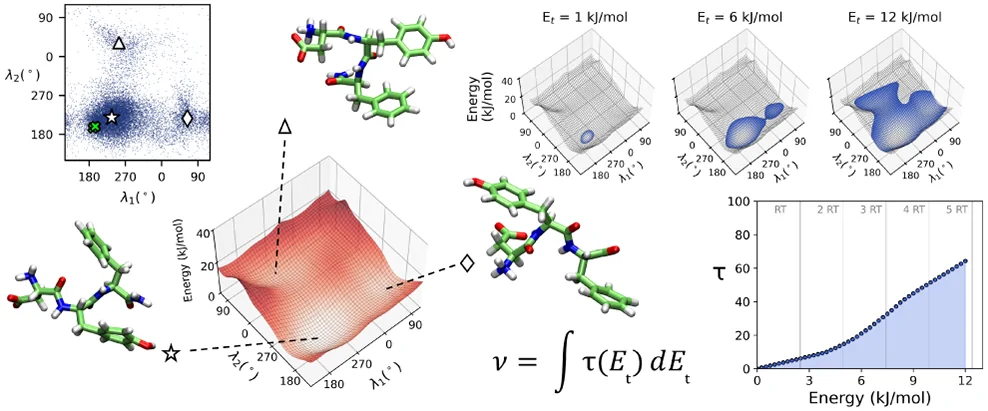

## Introduction

Peptides can be used as building blocks for the synthesis of a diverse range of supramolecular materials, including hydrogels,^1–3^ dynamic and porous crystal networks,^4^ glasses,^5,6^ and biomolecular condensates through liquid–liquid phase separation (LLPS).^7–9^ These properties can be leveraged for diverse functions, including drying-induced biomolecule encapsulation;^10^ the sequestration of rare-earth metals from aqueous solution;^11^ reaction mediation via LLPS;^12–14^ emulsification;^15–17^ or as drug molecule carriers or excipients,^18–21^ *etc*. The biodegradable and renewable nature of peptides^22,23^ is also advantageous for the design of material life cycles.^24^ However, the major barrier (and opportunity) to the widespread adoption of peptide-based materials remains the largely unsolved problem of rationally connecting a discrete amino acid sequence to desired material properties.

Due to the diversity of noncovalent interactions accessible within the vast supramolecular space available through combinations of the 20 canonical amino acids, each with their specific stabilities, interaction distances, and environmental dependencies, peptide materials may respond strongly to their environmental context, e.g., ionic strength, temperature, pH, and humidity.^16,25–27^ As a modular platform for materials chemistry and beyond, peptides are therefore an attractive class of molecules because their sequence space scales as 20^n^ (e.g., n = 3 gives 8,000 tripeptides), meaning a rich and context-dependent noncovalent chemical space^28^ can be realized with relatively short sequence lengths.

In an effort to address this challenge, researchers, including ourselves, leverage a combination of theory and experiment.^29–32^ Coarse-grained molecular dynamics (CG-MD) simulations play a prominent role in this area,^33–35^ particularly in the efficient screening of large peptide sequence spaces,^36–40^ which can be further enhanced through the adoption of data-driven and machine-learning-based approaches.^41–45^ To date, these efforts are discovery-based and, in some cases, generative, where demonstrated examples or newly proposed sequences are derived from correlation and are not yet designed on physical chemistry-based understanding, which is important for the ultimate goal of developing a universal assembly code for peptide materials. The formulation of a theory-informed, rational framework for *de novo* design remains an outstanding challenge. An important consideration in developing such a framework is accurately capturing the impact of peptide sequence and, more broadly, structural and sequence isomerism on phase properties. Often, this feature is de-emphasized when peptides are described in an additive sense, e.g., by representing their hydrophobicity as the sum of their amino acid LogPs.^42^ Clearly, this approach is insufficient, as several reports have shown striking differences in solubility or supramolecular self-assembly for isomeric peptide sequences.^25,46^ A connecting thread through these reports is side-chain-induced molecular conformation, which is an inherently sequence-specific property, determined by the relative location of side-chain functionality along a peptide backbone. This insight is already established in the context of heterochiral peptides, where subtle changes in conformation introduced by rearrangements of side-chain branching (heterochirality) can have dramatic impacts on resultant material properties.^47,48^

For example, Marchesan and coworkers theoretically evaluated the conformational free energy landscapes of the heterochiral sequence isomers V^L^F^D^ and F^D^V^L^. These were found to respectively be steep and shallow, which was correlated with their tendencies to form crystals directly (V^L^F^D^) or via an intermediate gel state (F^D^V^L^).^49^ We recently reported on the remarkably different solubilities of the hydrophobic I/L series of dipeptides (e.g., 0.1 and 0.66 mol L^−1^ for LL and II, respectively).. Using all-atom molecular dynamics (AA-MD) simulations, we observed that this can be explained through discrete differences in the conformational ensembles sampled by each sequence, showing that conformer selection can significantly impact bulk material properties.^26^

Extending to tripeptides, Gazit and coworkers reported that the tripeptide AFA has significantly higher solubility compared to its sequence isomers AAF and FAA (6- and 15-fold, respectively). Detailed investigations using MD simulations revealed differences in molecular conformations that modulate intermolecular interactions and self-assembly.^46^ A previous example from our laboratory was the six C-terminal amidated D/Y/F tripeptides (DYF, DFY, FDY, FYD, YDF, and YFD), for which we found that the position of aspartic acid (D) influenced their supramolecular assembly behaviors in buffered aqueous media.^50^ Specifically, the XDX tripeptides (FDY and YDF) remained soluble at 20 mM in phosphate buffer, while the DXX (DYF and DFY) and XXD (YFD and FYD) sequences formed ordered or disordered aggregates (YFD, DFY, and DYF formed self-supporting hydrogels or crystalline fibers, and FYD formed an amorphous precipitate). More recently, we also reported differential crystallization kinetics for the PXW and PXF tripeptides (X = F or W). Here, the PXW sequences spontaneously formed crystals while the PXF sequences remained in solution as supramolecular dispersions.^51^ In this case, analysis of single-molecule conformational ensembles modeled using MD simulations revealed a clear correlation between these different behaviors and the numbers of accessible conformational minima within 1 kT of the global minimum, which was taken as a proxy for conformational diversity. Specifically, the PXW sequences were found to have more accessible conformational minima within this cut off (2 in both PFW and PWW) compared to the PXF tripeptides (0 in PFF or 1 in PWF). Together, these reports suggest that an understanding of a peptide’s conformational ensemble could be leveraged to connect encoded sequence with solubility and emergent material properties.

In this work, we use AA-MD simulations to assess whether the single-molecule conformational ensemble of tripeptides is indeed informative and potentially predictive of peptide phase properties. To do so, we focused on two previously reported systems: the six amidated D/Y/F tripeptides and FFD. We hypothesized, and observed, that the degree of conformational accessibility within single-molecule free-energy landscapes (FELs) are predictive of a peptides’ tendency to be soluble and form supramolecular dispersions, or undergo self-assembly. We introduce a novel method to characterize FELs. Additionally, we evaluate the influence of two water models and sequence ionization states on conformational sampling. The analysis technique introduced here, and the insights described, are an advancement toward the accurate evaluation of peptide material properties based on amino acid sequence, which is an important step toward rational material design.

## Methods and Theory

### All Atom Molecular Dynamics (AA-MD)

#### System Construction

Peptide structures were generated using PeptideBuilder^53^ and processed using the GROMACS tool *gmx* pdb2gmx to make a topology file. The peptides were modeled using the Charmm36m (C36m) force field[Bibr ref54] (2022 distribution), and water was modeled using either the standard (sTIP3P^55^) or Charmm-modified (mTIP3P^56^) model. For the D/Y/F tripeptides, the N-terminus was protonated (NH_3_^+^), aspartic acid was deprotonated (COO^–^) and the C-terminus was amidated (−NH_2_). For FFD, the tested ionization states are summarized in Table S1. One tripeptide was randomly inserted into a 4 × 4 × 4 nm^3^ box for single-molecule simulations, and for multimolecule simulations, 70 molecules were randomly inserted into a 7 × 7 × 7 nm^3^ box. Initial solvation was done using a pre-equilibrated SPC water box, and where required, sodium or chloride ions were added with *gmx genions* to neutralize charges. All preparation was done using GROMACS 2022.5.^57^ Production simulations used versions 2022.5 or 2025.3-smp.

#### Nonbonded Interactions

At all stages, van der Waals (vdW) interactions were calculated using a cutoff distance of 1.2 nm, and a force-switch modifier was applied from 1.0 nm to smoothly switch the interaction energy to zero at the cutoff. Short-range electrostatics were calculated below a cutoff of 1.2 nm, beyond which long-range electrostatic interactions were treated using Particle Mesh Ewald (PME) electrostatics, with a Fourier grid spacing of 0.16 nm. A potential-shift modifier was also applied to the direct-space Coulombic potential as required for use of the Verlet cutoff scheme.

#### Minimization, Equilibration, and Production Simulations

Each system was initially minimized using the steepest descent algorithm (integrator = steep) for 50,000 steps to remove packing conflicts, with an energy tolerance of 1000 kJ mol^–1^ nm^–1^. Short (100 ps) NVT and NPT (100 ps, P = 1 bar, tau-p = 2 ps, barostat = Berendsen, β = 4.5 × 10^–5^ bar^–1^, coupling = isotropic) equilibrations were carried out while imposing positional restraints on the peptide(s) to allow water to equilibrate (integrator = leapfrog, ts = 2 fs). In the NVT preparation step, velocities were generated according to a Maxwell distribution at temperature of 298 K. This temperature was maintained at all simulation steps using the velocity-rescaling thermostat, with peptides and nonpeptides (water and ions) as separate coupling groups (tau-t = 0.1 ps). The LINCS algorithm^58^ was employed to constrain hydrogen bonds. Production simulations were done in the NPT ensemble using the Parrinello–Rahman barostat (β = 4.5 × 10^–5^ bar^–1^, coupling = isotropic). 250 and 500 ns trajectories were simulated for the single-molecule simulations, in duplicate and triplicate, respectively, and the multimolecule simulations were simulated for 500 ns in duplicate. To assess whether ensemble selection influenced the results, the different FFD ionization states were also simulated in the canonical ensemble (NVT). Here, the same preparation procedure was followed (i.e., minimization, NVT and NPT equilibrations), with the only difference being that no pressure coupling was used in the production trajectory. Using the characterization methodology described below, it was found that the method is not ensemble-dependent (Tables S10 and S15, Figure S6). These settings were adapted from the Justin A. Lemkul GROMACS tutorials.^59^

### Conformational Free Energy Landscape Generation and Characterization

Tripeptide λ torsions were measured between the center of mass (COM) positions of backbone (BB) and side chain (SC) fragments (Figure 1A) using code developed in our laboratory, which utilized the Python package MDAnalysis.^60^ Torsional angles are continuous within 360°; however it was found that there was essentially no sampling at λ ≈ 120°, which we interpret as being a consequence of side chain chirality and steric restraints. Consequently, all angular data λ < 120° were shifted by 360° into the angular range [120°, 480°). For ease of interpretation, tick labels in all figures have been relabeled 0° to 360°. For a tripeptide there are two λ torsions, λ_1_ and λ_2_, corresponding to the torsions between side chains 1–2 and 2–3, respectively, which generate a rich torsion space (Figure 1B). This torsion space can then be converted into an FEL (Figure 1C) through a two-step process: first, a probability density function (PDF) is made using bivariate isotropic Gaussian kernel density estimation (KDE; eq 1).^61,62^

1 $$p\left(\lambda_{1},\lambda_{2}\right)=\frac{1}{2\pi nh^{2}}\overset{n}{\underset{i=1}{\sum }}\exp\left(-\frac{{\left(\lambda_{1}-\lambda_{1i}\right)}^{2}+{\left(\lambda_{2}-\lambda_{2i}\right)}^{2}}{2h^{2}}\right)$$

**Figure 1:**
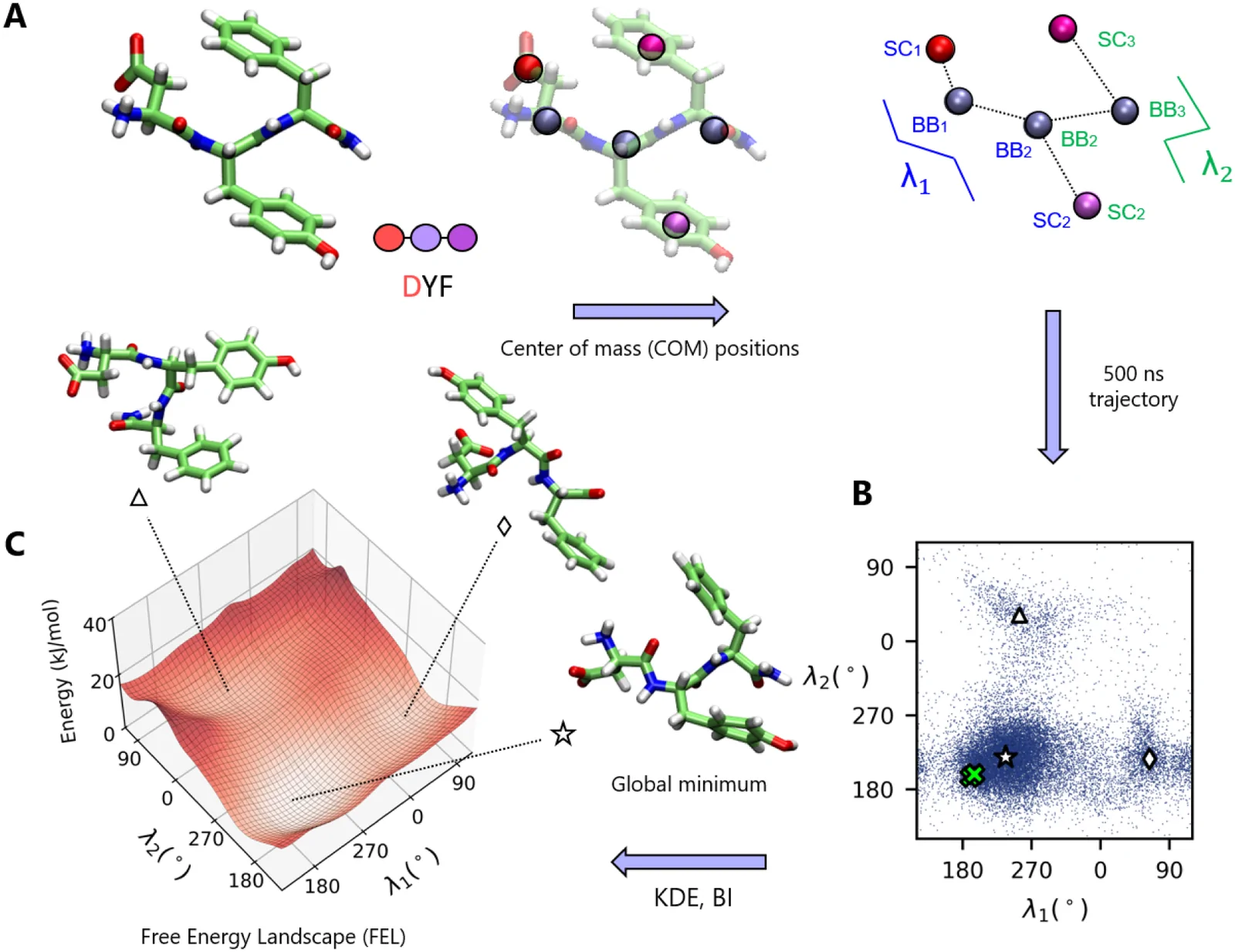
Conformational analysis workflow for tripeptides. (A) Simplification of representative tripeptide DYF-NH_2_ into backbone (BB) and side-chain (SC) center-of-mass (COM) positions, with definition of torsional collective variables λ_1_ and λ_2_. (B) Torsions map for λ_1_ and λ_2_ after 500 ns simulation in sTIP3P water, showing regions of high sampling as those with dense color; green crosses correspond to the crystal structure conformation of DYF (CCDC deposition number: 1539905), and the global (☆) and local (◊ and △) minimum energy positions found in the 3D FEL in (C). (C) Free energy landscape (FEL) obtained from torsional data in (B) after kernel density estimation (KDE) and Boltzmann inversion (BI). Conformers shown in the inset are those closest to the minimum energy coordinates found within the FEL, and the global minimum in this case corresponds to a conformation where amino acids are presented at alternating sides of the backbone, as found in the crystal structure (Figure S5C). All structures were rendered using visual molecular dynamics (VMD) software.^52^

Where *n* corresponds to the number of data points and *h* to the bandwidth. We evaluated a range of bandwidths, starting from the Scott’s rule value (eq 2) and increasing in increments of 0.05 (i.e., [0.1919, 0.3919]).

2 $$h=n^{-\frac{1}{d+4}}$$

Where *n* corresponds to the number of data points and *d* is the dimensionality of the data (i.e., *n* = 20,001 frames, *d* = 2 torsional dimensions, gives *h* = 0.191937). A value of *h* = 0.291935 was chosen to generate the FELs described in the main text (Tables S8–S10, S15); this value falls within a range that gave an intermediate number of minima (where an overly rough surface gives too many and an overly smooth one too few; Figure S2), and this was confirmed by qualitative inspection of raw data and generated FELs (Figure S3). Furthermore, we found that the relative ordering of the amidated D/Y/F tripeptides with respect to ν was independent of bandwidth selection, indicating that the method is robust with respect to this empirically selected tuning parameter (Figure S4).

After the PDF was constructed, an angular grid with 1° spacing (360 × 360 = 129,600 total, *N*_total_) was made to evaluate the probability density at each grid point. Then, the relative energy at each grid point was evaluated via Boltzmann Inversion (BI, eq 3).

3 $$U\left(\lambda_{1},\lambda_{2}\right)=-RT\ln\left(p(\lambda_{1},\lambda_{2}\right))$$

Where *R* is the ideal gas constant, *T* is the temperature (T = 298 K), and *p*(*λ*_1_,*λ*_2_) is the probability density estimate at a given coordinate [*λ*_1_,*λ*_2_) in the grid. A small constant probability (ε = 1 × 10^–12^) was added to all positions to avoid numerical instabilities for unsampled grid points. Additionally, to facilitate minima processing, we assigned np.nan to all positions with energy >40 kJ/mol, effectively deleting them, as all such positions corresponded to regions of low sampling and so have lower accuracy. After normalization, such that the global minimum grid point had energy 0 kJ/mol, we utilized the SciPy package ndimage^63^ to identify positions within the surface corresponding to energy minimum, defined as positions about which all immediate neighbors are greater in energy.

To evaluate the thermal conformational accessibility of the FEL, we then quantified the percentage of points within the grid, τ, that were less than or equal to an iteratively increasing threshold (*E*_t_, initially 0 kJ/mol), within the interval 0 to 12 kJ/mol (0 to ∼5 kT on a per-molecule basis), in increments of 0.25 kJ/mol (Figure 3A, 3B, eq 4).

4 $$\tau \left(E_{t}\right)=100\%\cdot \frac{\sum N\left(E\leq E_{t}\right)}{N_{\mathrm{total}}}$$

We then integrated this relationship within the defined interval using the NumPy^64^ implementation of the trapezoid rule (eq 5) to find the area under the curve, ν, (Figure 3B,C).

$$\nu =\int_{0\,\;\mathrm{kJ}/\mathrm{mol}}^{12\,\;\mathrm{kJ}/\mathrm{mol}}\tau \left(E_{t}\right)dE_{t}$$ 5

Given the angular nature of the λ torsions and associated periodicity, a bivariate von Mises distribution would, in principle, be the preferred density estimator, with the data projected onto a torus.^65^ Currently, however, there is no readily implemented library for these calculations, and this represents a direction for future method development. The consequence of this would be that the accuracy of probability (and energy) estimates at the FEL edges may be reduced. To evaluate the impact of this on the reported trends between sequences, we constructed a PDF using a 2D-histogram on the basis that this would be less susceptible to edge effects. To do this, we used the NumPy function histogram2d over the interval [120°, 480°] using a bin spacing of 10° (i.e., 36 × 36 bins total). It was found that the sequence-dependent differences in ν for the D/Y/F tripeptides estimated using the Gaussian KDE method were also obtained using this histogram-based approach, as were the water model-dependent differences described below (Figures S7 – S10). The surfaces derived using this method were rougher compared to those obtained by KDE, and significant regions, where bins were empty or had high energies (>40 kJ/mol), were removed from the surface using the method outlined above (Figures S11–S13).

### Water Model Evaluation

During the development of this method, we found that the selection of the water model influenced both the intrinsic conformational ensemble of peptides (i.e., FEL steepness and *ν* values) and the aggregation of peptides in multimolecule simulations, as we have reported previously.^51^ The difference between the sTIP3P and mTIP3P water models is the presence of a Lennard-Jones term on the water hydrogen atoms for the mTIP3P version (σ_*HT*_ = 0.04 nm and *ε*_*HT*_ = 0.1925 kJ/mol), which were added to avoid singularities within integral equations,^56^ whereas in the standard model these terms are set to zero.^55^

To quantify the effect of this difference, we first evaluated the number of peptide-water hydrogen-bonding interactions for the single-molecule trajectories using *gmx hbonds* (default settings) as a proxy for interaction strength. The average number of hydrogen bonds ⟨*n*_*HB*_⟩ was then evaluated for the final segment of the trajectory and weighted for the number of water molecules within the simulation box (*N*_*w*_, Table S2) according to eq 6.

$$\left\langle n_{HB}\right\rangle =\frac{1}{N}\frac{1}{N_{w}}\underset{t}{\sum }N_{HB}\left(t\right),\mathrm{for}\,\;t\in [t_{\mathrm{initial}},t_{\mathrm{final}}]\;ns$$ 6

where *N*_*HB*_(*t*) is the number of hydrogen bonds at time *t*, and *N* is the duration in ns of the final trajectory segment. For 250 ns the final trajectories segment was [200, 250] ns, and for the 500 ns trajectories it was [400, 500] ns. Means and standard deviations are provided in Tables S3–S6. For a given sequence and simulation time, the relative free energy difference of peptide-water hydrogen bonding between the water models was calculated according to eq 7.

7 $$\Delta G_{HB}=-RT\ln\left(\frac{{\langle n_{HB}\rangle }_{mTIP3P}}{{\langle n_{HB}\rangle }_{sTIP3P}}\right)$$

where ⟨*n*_*HB*_⟩_*mTIP3P*_ and ⟨*n*_*HB*_⟩_*sTIP3P*_ correspond to the weighted and time-averaged number of peptide-water hydrogen bonds, R corresponds to the ideal gas constant, and *T* is the temperature (298 K) (Table S7). We then evaluated ν for the 500 ns single-molecule amidated D/Y/F trajectories using the method already described for simulations using both water models to assess the impact of this effect on the resultant conformational FELs (Figure 3C, 3D and Tables S8, S9).

As a third analysis, we performed multimolecule simulations of the D/Y/F tripeptides to assess the impact of both water models on molecular aggregation. Here, the solvent-accessible surface area (SASA) of the peptides was measured on periodic-boundary-corrected trajectories using *gmx sasa,* with default settings. Aggregation propensities (APs) were then evaluated using these results. The AP score is defined as the ratio of solvent accessible surface area (SASA) between the peptides in the initial state *SASA*_initial_, versus the measurement at time *t* in the final section of the trajectory, *SASA*_*t*_, exceeding 1.0 for aggregated states. This is enumerated as an average over the final 100 ns, where the system is deemed to have equilibrated, eq 8.

$$\left\langle AP\right\rangle =\frac{1}{N}\underset{t}{\sum }\frac{SASA_{\mathrm{initial}}}{SASA_{t}},\mathrm{for}t\in [400,500]\;ns$$ 8

where *N* is the duration of the final section of the trajectory (*N* = 100 ns). Mean and standard deviation values were calculated based on the mean AP scores from the tested interval and evaluated from duplicate trajectories for both mTIP3P and sTIP3P water models (Tables S11, S12).

## Results and Discussion

We hypothesized that the ability of peptides to undergo self-assembly is correlated with conformational accessibility: this property corresponds to the number of unique, thermally accessible conformation states, which in turn should be informative of the degree to which peptides can undergo conformational rearrangement as they organize into higher-order structures during assembly. On this basis, self-assembling sequences should have low activation energies of conformational change; conversely, those that do not assemble but instead remain soluble or loosely/dynamically interact through reversible contacts that do not impact their conformations should have concomitantly high energy barriers. To evaluate this hypothesis, MD simulations were used to sample the conformational ensembles of the amidated D/Y/F tripeptides that we previously characterized,^50^ with the conjecture that conformational accessibility within an ensemble can act as an indirect measure of the activation energy of conformation change which, in turn, is informative of self-assembly propensity. Specifically, an assembling sequence should have a shallow free energy landscape, meaning broad conformational accessibility corresponding to low energy barriers of conformation change, and a nonphase-separating sequence should have a steep landscape, with limited conformational accessibility and high energy barriers.

### Tripeptide Side Chain Conformations as a Collective Variable Can Distinguish Differences between Sequence Isomers

To measure tripeptide conformations and explicitly connect them to discrete amino acid sequences, we first chose to simplify a peptide into its constituent parts: backbone (BB) and side-chain (SC) units, and represent them as positions in space defined by their centers of mass (COM). Using these positions, we define and measure a collective variable, the torsional angle λ between consecutive side chains relative to the peptide backbone (i.e., SC_i_ – BB_i_ – BB_i+1_ – SC_i+1_) over the course of an MD simulation (Figure 1A). This technique extends on our previously reported method, which tracked aromatic side chains only,^50^ and is now generalized to focus on the relative positions of all three side chains with respect to the backbone. The FEL generation scheme presented here and outlined in the Methods (Figure 1B, 1C) is the same as was used earlier to analyze the PXX tripeptides.^51^ In those sequences, the N-terminal proline imidazole rings enforced conformational rigidity, so we first decided to evaluate whether this collective variable was capable of distinguishing differences between the more flexible amidated D/Y/F tripeptides.^50^

Qualitative inspection of the λ_1_/λ_2_ torsional data confirmed this capability. For the XDX sequences, most of the sampling occurs within the region [120° < λ_1_ < 315°, 180° < λ_2_ < 270°], and, to a lesser extent, [225° < λ_1_ < 280°, 270° < λ_2_ < 0°]. The major population corresponds to structures with consecutive side chains projecting away from one another in an alternating *anti* conformation (Figure 2A, 2C). This can be understood as being a consequence of the lack of favorable noncovalent interactions between the 1–2 and 2–3 side chains (i.e., aromatic (F/Y) – charged hydrophilic (D) interactions) and a more favorable 1–3 contact (i.e., F/Y – Y/F). The DXX and XXD tripeptides also display this alternating *anti* conformation, and for DYF this sampling domain is close to the λ_1_/λ_2_ torsions associated with its crystal structure conformations (Figure 2A, green crosses).^50^ For these sequences, there is also an additional subpopulation of conformers that adopt either a 1–2 or 2–3 *syn* conformation where aromatic side chains are orientated toward one another through complementary F/Y – Y/F interactions (XXD [120° < λ_1_ < 90°, 180° < λ_2_ < 270°] and DXX [120° < λ_1_ < 0°, 0° < λ_2_ < 90°], respectively; Figure 2A). In the case of YFD, this 1–2 *syn* arrangement is also present in its crystal structure conformation (Figure 2A, green crosses). Together, the λ_1_/λ_2_ collective variables indicate that the XDX sequences have a more unimodal conformational ensemble compared to the XXD and DXX sequences, which are more multimodal.

**Figure 2:**
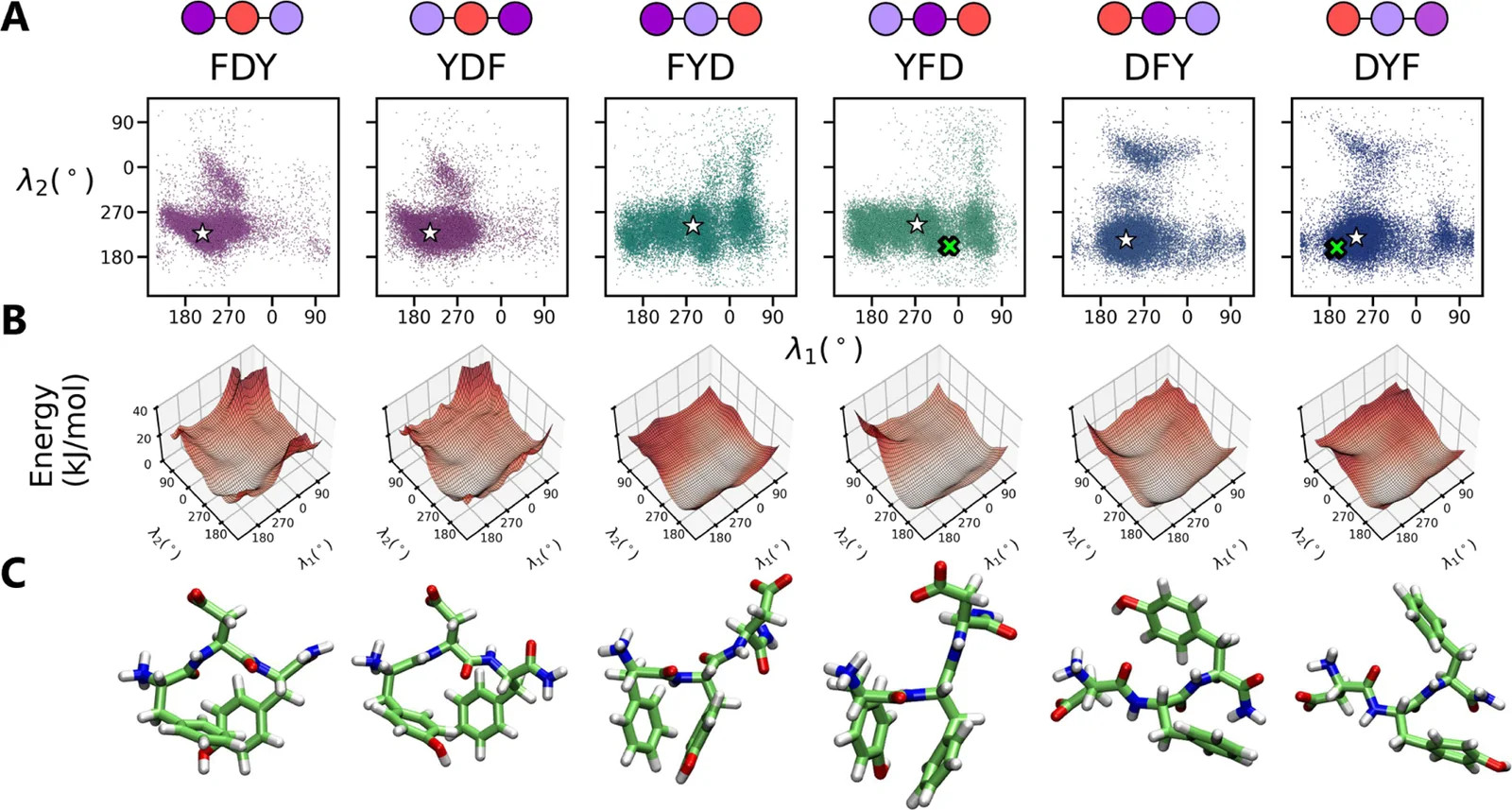
Sequence dependence of D/Y/F tripeptide conformational sampling using sTIP3P water. (A) Single-molecule conformational sampling in λ_1_ and λ_2_ torsions for the D/Y/F tripeptides, where each plot shows 20,001 data points derived from 500 ns trajectories; green crosses for DYF and YFD correspond to crystal structure torsional angles (CCDC deposition numbers: 1539905 and 1539906, respectively); (B) free energy landscapes (FELs) obtained via Boltzmann inversion of the probability density function (PDF) constructed using λ_1_ and λ_2_ for amidated D/Y/F tripeptides; (C) closest conformer to the global minimum within conformational FEL, the position of which is indicated by (☆) in panel (A); for XDX and DXX sequences, this corresponds to an alternating arrangement, while for the XXD sequences a less structured 1–2 aromatic self-stack is adopted. These conformations are similar to those identified in our earlier work,^50^ though for the XXD sequence the resemblance is reduced. This difference is likely due to the conformer selection criteria; the present method accounts for both 1–2 and 2–3 torsions, whereas our earlier work was focused on the interaromatic torsional angle only. DYF has a qualitative structural similarity to the crystal structure conformations we reported previously (Figure S5C). All structures were rendered using VMD.^52^

### Thermal Conformational Accessibility Measured for Single Molecule Tripeptide FELs Correlates with Reported Assembly Propensity

We generated FELs for each tripeptide using the scheme described in the Methods section (i.e., PDF generation using KDE followed by Boltzmann inversion) and observed qualitatively that the smoothness increased as XDX < DXX < XXD (Figure 2B), in agreement with the unimodal/multimodal structure of the initial sample distribution (Figure 2A). To quantify this difference, we applied our thermal conformational accessibility evaluation method (see Methods) to estimate ν values. It was found that this method was able to distinguish differences in FEL curvature, for example, the tripeptide YFD-NH_2_ shows a much faster increase in conformational accessibility with respect to threshold energy versus FDY-NH_2_, with DYF-NH_2_ as an intermediate between these extremes (Figure 3A, 3B). For the six sequences, a quantitative increase in ν was found in agreement with the qualitative ordering of FEL smoothness, and this trend was consistent across both water models (Figure 3C, 3D). Using sTIP3P water, this was: ν_*XDX*_ ≈ 150–190, ν_*DXX*_ ≈ 240–300, and ν_*XXD*_ ≈ 400–410% kJ/mol (Table S9). These differences correlate well with the observed supramolecular behaviors, where the XDX sequences with steep FELs (low ν) do not self-assemble and remain soluble, while the DXX and XXD sequences with shallow FELs (high ν) show aggregation, fibrillization, or crystallization. This result supports our hypothesis that sequences with higher levels of conformational accessibility are more likely to undergo self-assembly.

**Figure 3:**
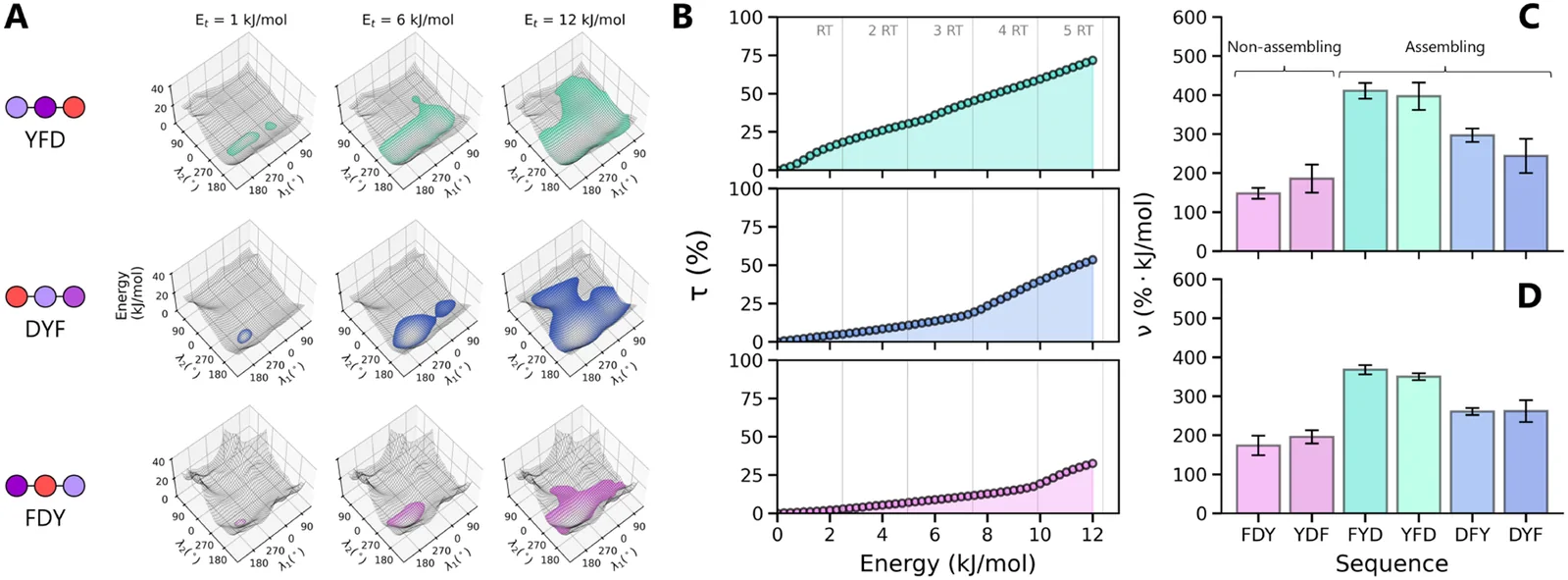
Illustration of free energy landscape (FEL) characterization by evaluation of thermal conformational accessibility. (A) Representative free energy landscape for amidated tripeptides YFD-NH_2_, DYF-NH_2_ and FDY-NH_2_ showing the extent of the accessible surface at ≤ 1, 6, and 12 kJ/mol. (B) Illustration of *τ* parameter versus the energy threshold *E*_t_ evaluated within the interval [0, 12] kJ/mol; this increases fastest for YFD, followed by DYF, and slowest for FDY. Triplicate mean FEL thermal conformational accessibility parameter *ν* for the amidated D/Y/F tripeptides, evaluated from simulations using the sTIP3P (C) and mTIP3P (D) water models; experimental assembly outcome is included in C.^50^ Error bars correspond to the standard deviation calculated from triplicate simulations.

### Water Model Selection Impacts the Conformational Ensemble and Aggregation Propensities of Tripeptides

We observed the same trend in thermal conformational accessibility for both sTIP3P and mTIP3P water models (Figure 3C, 3D and Tables S8, S9), however, the difference between sequences was more pronounced in the case of sTIP3P water. To understand this effect, we evaluated the relative free energy of the peptide-water interaction using the number of peptide-water hydrogen bonds as a proxy for this (see Methods). The relative free energy differences between mTIP3P and sTIP3P were found to be −0.048 ± 0.022 kJ/mol and −0.065 ± 0.028 kJ/mol for the 250 and 500 ns trajectories, respectively (Table S7). The close agreement between these measurements, within 1 σ of each other, indicates that the simulation duration does not strongly impact this result. These measurements evidence that the peptide-water interaction is more favorable for mTIP3P water compared to sTIP3P.

Water model selection is also known to influence peptide-peptide aggregation propensity (AP) scores; for example, this is observed in the context of CG simulations of short peptides.^66^ We recently found that this had a dramatic impact on the AP scores for the PXX tripeptides^51^ and sought to evaluate this effect for the amidated D/Y/F tripeptides. It was found that the mTIP3P water model led to a systematic reduction in AP compared to the sTIP3P model (Figure S1, Tables S11 and S12). Here, the average AP over all six sequences was x̅_*AP*_*mTIP3P*_ = 1.815 ± 0.193 and x̅_*AP*_*sTIP3P*_ = 2.490 ± 0.149 for the mTIP3P and sTIP3P models, respectively. This can be explained on the basis that peptide-water interactions are more favorable in mTIP3P water and so aggregative peptide-peptide interactions are less stabilizing compared to the sTIP3P case. This is in agreement with previous studies using the earlier C36 force field, which also showed that mTIP3P water better stabilized unfolded peptides and protein states.^67,68^

On the basis of these experiments, we can understand the observed differences in both the discrete conformational ensemble of tripeptides and their propensity to aggregate in terms of the relative favorability of peptide-water interactions. Moving forward, on the basis of these results, sTIP3P water is the preferred model for differentiating sequence isomers. However, given the small sequence space in our present study, this consideration remains important and should be evaluated when possible in future studies.

### Tripeptide Conformational Ensembles are Ionization State Dependent

For the C-terminal amidated D/Y/F tripeptides, all simulations were done in the charge-neutral, zwitterionic form. This ionization state was selected because it is the state observed in our reported crystal structures of DYF and YFD.^50^ More broadly, we expect that the neutral form of the peptide (zwitterionic or uncharged) is likely the assembly-relevant species, as it alleviates intermolecular electrostatic repulsions between sequences with a net charge. There is good evidence of this, in particular, for assembling peptide molecules with a single carboxylic acid (such as the N-terminal-capped aromatic peptide amphiples^69^), In this molecular class a substantial apparent shift in *pK*_a_ can occur, leading to COO^–^ protonation, with the neutral species becoming dominant in the solid/gel state.^70,71^ As our method relies on single-molecule simulations of a fully solvated peptide, the default ionization state corresponds to that expected at pH 7. However, the assembling species may protonate or deprotonate to adopt a charge-neutral form upon assembly, and we therefore sought to evaluate how ionization states influence the underlying conformational ensemble.

We chose the tripeptide FFD as a model system for this evaluation for several reasons: first, it has a close structural similarity to YFD and is also a fibril-forming gelator;^2,15,72^ second, its conformation in the crystalline state is qualitatively very similar to that of YFD (i.e., a 1–2 aromatic self-arrangement and an N-terminus-to-Asp salt bridge; Figures S5A, S5B) and, lastly, it displays pH-dependent self-assembly (i.e., pH ≤ 5), requiring a major population of the zwitterionic species for self-assembly in which the C-terminus is protonated, as has already been rigorously characterized by Marchesan and coworkers.^2^

Thus, we simulated a single molecule of FFD in all four charge states that occur within the pH range 0 – 14—i.e., FFD­(+1), FFD(0), FFD(−1), and FFD(−2) (Table S1)—using the same methodology as already described, with sTIP3P water. Qualitative inspection of both the raw λ_1_/λ_2_ torsional sampling (Figure 4A) and the smoothness of generated FELs (Figure 4B) revealed a contraction in conformational diversity, following the trend FFD(0) > FFD­(+1) > FFD(−1) > FFD(−2). Evaluation of ν gave quantitative agreement with this ordering (Figure 4D, Table S10). Most remarkable was the low value obtained for FFD(−2), with ν_*FFD*(−2)_ = 130 ± 6 kJ/mol, which is below those found for nonassembling sequences FDY and YDF (Figure 3C). These observations confirmed that the selection of the peptide ionization state does indeed impact the sampled conformational ensemble.

**Figure 4:**
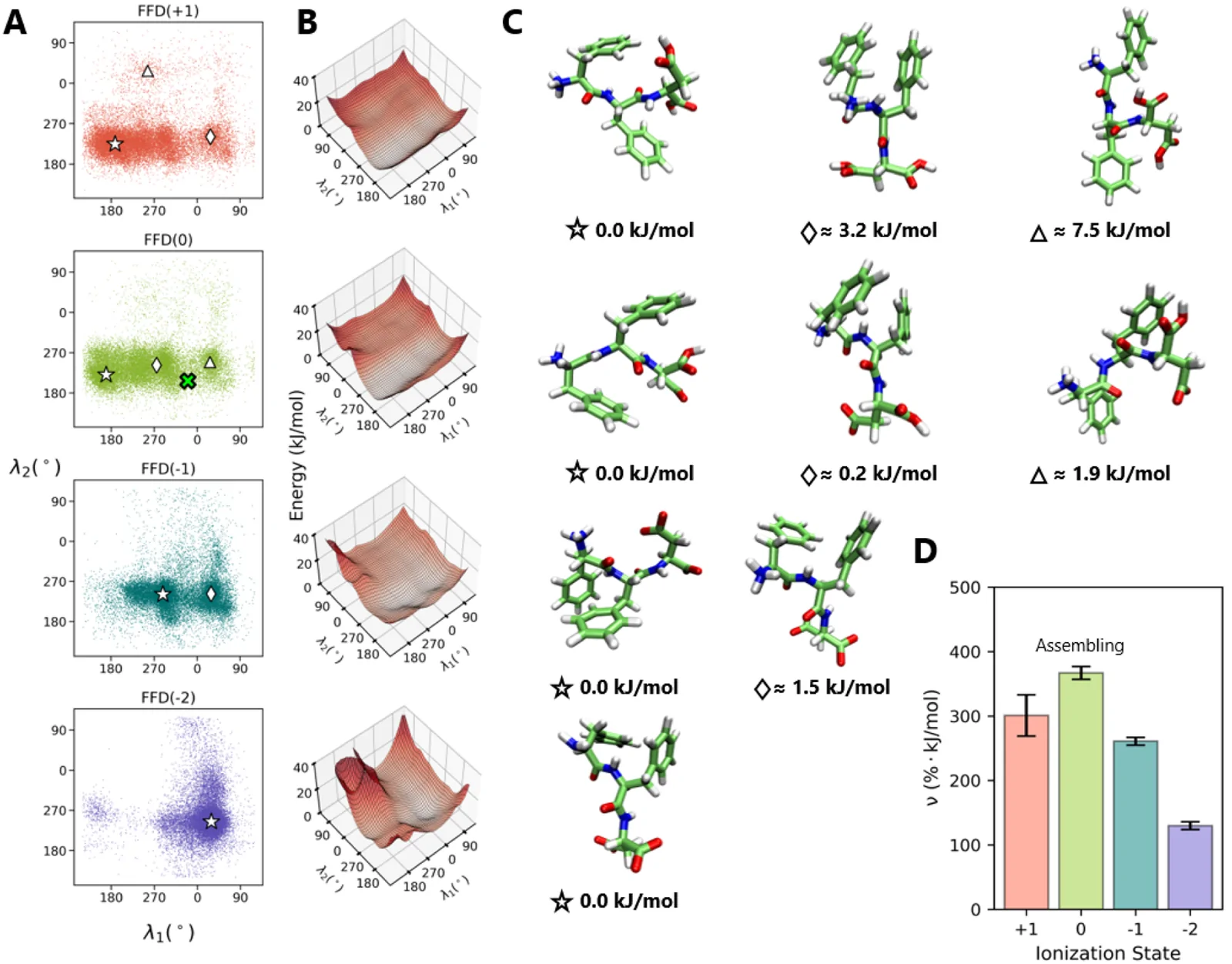
The influence of ionization state on the conformational ensemble of FFD. (A) Single-molecule conformational sampling in λ_1_ and λ_2_ torsions for each ionization state of FFD (+1, 0, −1, and −2), each plot shows 20,001 data points derived from 500 ns trajectories; the global (☆) and local (◊ and △) minimum-energy positions found in the 3D FEL; the green cross for FFD(0) corresponds to the λ_1_ and λ_2_ torsions of the FFD crystal structure. (B) Free energy landscapes (FELs) obtained via Boltzmann inversion of probability density function (PDF) constructed using λ_1_ and λ_2_ for each FFD species. (C) Conformational minima with energy <8 kJ/mol (∼3 RT) for FFD tripeptides in each ionization state showing intramolecular interactions, including π-π self-stacking, salt-bridge formation, and hydrophobic pockets formed between protonated carboxylates and aromatic side chains; their positions within the λ_1_ and λ_2_ landscape are indicated by the symbols ☆, ◊, and △. (D) Triplicate mean FEL thermal conformational accessibility parameter *ν* for FFD in each ionization state, evaluated within the energy interval [0, 12] kJ/mol. Error bars correspond to the standard deviation calculated from triplicate simulations. All structures were rendered using VMD.^52^

To understand these findings in more detail, we investigated the underlying intramolecular interactions between the side chains and termini. Three main modalities emerged from visual inspection: 1–2 aromatic self-stacking, hydrophobic pocket formation between aromatic side chains and protonated carboxyl groups, and salt bridging between the N-terminus and deprotonated carboxylates (Asp or C-terminus). Like the amidated XXD sequences, the most sampled degrees of conformational freedom exist in the 1–2 interaction, as demonstrated by the broad sampling of 0° < λ_1_ < 360° (Figure 4A, 4B). FFD­(+1) samples three major conformation states, an alternating [λ_1_ ≈ 180°, λ_2_ ≈ 180°], 1–2 self-stacked [λ_1_ ≈ 0°, λ_2_ ≈ 225°], and 2–3 self-stacked arrangement [λ_1_ ≈ 270°, λ_2_ ≈ 0°], where there is an F(2)-D(3) hydrophobic pocket (Figure 4C, first row). In FFD(0), the alternating and 1–2 self-stacked structures are conserved, whereas the intra side chain 2–3 contact is lost (through D(3) ionization) and replaced by an F(2)-protonated C-terminus hydrophobic pocket (Figure 4C, second row, ≈1.9 kJ/mol conformer), resulting in a diverse conformational ensemble for this state. For FFD(−1) the 2–3 interaction is repulsive both from the D(3) and deprotonated C-terminus; consequently, the 1–2 self-stacked and alternating states become more stable, and conformational diversity in the λ_1_ dimension is reduced (Figure 4C, third row). Lastly, for FFD(−2) the loss of intramolecular interactions is most pronounced, and the 1–2 aromatic self-stack dominates, with D(3) and the C-terminus projected into solution (Figure 4C, fourth row). This last result, and the loss of dynamicity in the λ_1_ torsion, indicates that N-terminal protonation contributes to conformational diversity in this dimension. Taken together, these results confirm that the conformational ensemble sampled by a peptide is dependent on the selected ionization state and that this can be rationalized in terms of intramolecular interactions within the sequence.

### Interpretation of Conformational Ensembles within a Mechanistic Model for Peptide Self-Assembly

Our present results are conceptually connected to the proposed, and increasingly described, liquid-liquid phase separation (LLPS)-mediated model of peptide self-assembly.^73–77^ In this model, long-range noncovalent interactions such as the hydrophobic effect and/or attractive electrostatics result in the formation of a low-order dense peptide phase in solution from which short-range noncovalent interactions nucleate the growth of higher-order structures.^75^ Such a modality of the initiation of fiber formation, from low-order, hydrophobicity-driven association to local order hydrogen-bonding-nucleated directional growth, is not peptide-specific and has been described in detail for other supramolecular assembling architectures using molecular modelling.^78^ This mechanistic model implies that the thermodynamic feasibility of self-assembly is connected to the energetics of conformational change and the degree of conformational accessibility within the ensemble. In the present interpretation of our reported correlation, we assert that dynamicity at infinite dilution informs, but does not directly measure, the energetics of reorganization within a dense phase. Applying this interpretation to the D/Y/F tripeptides, we might expect that they are equally capable of forming a dense phase (based on similar or the same intrinsic hydrophobicity), but only those with sufficient scope to change conformation can propagate into self-assembled states. This would account for the similar AP scores observed for these sequences using the sTIP3P water model (Figure S1). We propose that the sequences that do not meet this criterion (i.e., XDX tripeptides) might instead form supramolecular dispersions; this is a solution state that we recently described for K/Y/W tripeptide isomers, characterized by a highly dynamic and interacting dense phase that does not undergo phase separation.^10^ In terms of LLPS-mediated self-assembly, such a state might be thought of as being kinetically trapped, although further work is required to explore these ideas in more detail.

### Outlook with Respect to the Wider Tripeptide Sequence Space

The tripeptides in the present study are constituted of a small number of amino acids to demonstrate the methodology for reported examples (i.e., Y, F, and D); however, the rich chemical functionality represented by the 20 canonical amino acids is expected to generate diverse conformational ensembles and emergent assembly behaviors. For example, highly multivalent amino acids, such as arginine (R), can engage in a diversity of noncovalent interactions,^28^ which will generate unique conformational ensembles. Recently, it was shown that side-chain branching at either the β- or γ- carbon position in the hydrophobic tripeptides FFF, LLL, and III controlled the degree of extended-conformation content within simulated assemblies. Here, single-molecule conformational analysis revealed that conformational restrictions present in β-branched III resulted in preorganization toward the extended state. In contrast, the γ-branched sequences FFF and LLL adopted both folded and extended conformations, arising from favorable intramolecular interactions between side chains, which resulted in more heterogeneous conformational ensembles in the aggregated state.^79^ More broadly, it is known that system-level changes in multivalency and chemical context, through the influence of ions, solvents, or ligand molecules, can change self-assembly outcomes.^80–83^ The connection between these influences, specific amino acid content, and conformation remains to be explored, though we anticipate that the present methodology may be applied to such questions.

## Conclusion

In this study, we described a sequence-encoded method for analyzing the conformational ensembles of seven tripeptides—six sequence isomers and the closely related sequence FFD. Our hypothesis was that the degree of conformational accessibility can act as an indirect measurement of activation energies of conformation change, which, in turn, is informative of a sequence’s self-assembly propensity. This hypothesis is connected to an interpretation of the LLPS-mediated model of peptide self-assembly, in which it is implied that conformational change occurs as peptides transition from a dense phase into higher-order structures.

We observed that for the amidated D/Y/F tripeptides, the sequences with the shallowest free energy landscapes, evaluated with respect to their conformational accessibility response to increasing thermal energy, correspond to self-assembling sequences in agreement with reported experimental observations and our hypothesis. An important consideration in these analyses, not captured explicitly by this technique, is the role of intermolecular peptide interactions. This is particularly relevant in the consideration of electrostatic effects that are both likely to restrain the initial formation of a dense phase (i.e., leading to peptide solubility) and/or give rise to *pK*_a_ shifts, where changes in ionization state can occur to facilitate self-assembly. The method described here does not explicitly account for such cooperative effects, but we propose that this can be accounted for by applying this analysis to the assembly-relevant ionization state (i.e., the electrostatically neutral form). We demonstrated this for the tripeptide FFD, where we showed that the sampled conformational ensemble is dependent on the ionization state as a consequence of the different intramolecular interactions that can occur.

Lastly, the simplification of a complex free-energy landscape into a single numerical parameter, ν, is an important outcome of this work as it can be readily integrated within data-driven approaches or could be used as a cheap prescreening technique in a complementary manner with multiscale methods (i.e., CG-MD). Furthermore, this parameter is implicitly sequence-encoded and at all steps can be intuitively rationalized in terms of intramolecular interactions. In the future, we plan to evaluate whether this approach and theoretical understanding can be generalized for the *de novo* design of tripeptide materials for functional applications.

## Supplementary Material



## Data Availability

The simulation data underlying this study, as well as the preparation scripts for those simulations, are available at: https://doi.org/10.5281/zenodo.20532575. The scripts and code used to analyze the simulation trajectories using the described method are available at: https://github.com/HamishSwanson/Molecular-conformation-as-a-determinant-of-self-assembly-in-sequence-isomeric-tripeptides

## Notes

Supporting Information: tables of simulation details (Tables S1 and S2); tabulated results of all plotted data (Tables S3–S15); supporting figures, including results and comparisons of measurements pertaining to the development and validation of the free-energy landscape (FEL) analysis method described in the main text (Figures S1–S13) (PDF)

## Abbreviations

AA: all-atom
AP: aggregation propensity
BB: backbone
BI: Boltzmann inversion
CG: coarse-grained
COM: center-of-mass
FEL: free energy landscape
KDE: kernel density estimation
MD: molecular dynamics
PDF: probability density function
SC: side chain
