# Supplementary material for: Molecular Conformation as a Determinant of Self-Assembly in Sequence-Isomeric Tripeptides
Source: J Phys Chem B. 2026 Oct 12;130(42):11190–201. doi: 10.1021/acs.jpcb.6c03754 (PMC13700000; doi:10.1021/acs.jpcb.6c03754)
Supplement: Supplementary file 1 [file jp6c03754_si_001.pdf]

Supporting Information for Publication:

**Molecular conformation as a determinant of self-assembly in sequence-isomeric tripeptides**

Zhuojun Li[1,2], Rein V. Ulijn[1,2,3] and Hamish W. A. Swanson[1]

[1] Advanced Science Research Center (ASRC) at the Graduate Center of the City University of New York, 85 St Nicholas Terrace, New York, 10031, USA.

[2] Department of Chemistry, Hunter College, City University of New York, 695 Park Avenue, New York, 10065, USA.

[3] Ph.D. Programs in Chemistry and Biochemistry, The Graduate Center of the City University of New York, New York, 10016, USA.

*Corresponding Authors: hamish.swanson05@gc.cuny.edu; rulijn@gc.cuny.edu

Table S1 – Summary of tested FFD ionization states

| Species | N-terminus | Asp(3) | C-terminus |
|---|---|---|---|
| FFD (+1) | protonated | protonated | protonated |
| FFD (0) | protonated | deprotonated | protonated |
| FFD (-1) | protonated | deprotonated | deprotonated |
| FFD (-2) | deprotonated | deprotonated | deprotonated |

Table S2 – Water molecule content for D/Y/F tripeptide simulations

| Sequence | mTIP3P | sTIP3P |
|---|---|---|
| FDY | 2142 | 2143 |
| YDF | 2141 | 2143 |
| FYD | 2144 | 2144 |
| YFD | 2141 | 2142 |
| DFY | 2144 | 2144 |
| DYF | 2141 | 2144 |

Table S3 – Average amidated D/Y/F tripeptide – water hydrogen bonds (mTIP3P, 250 ns)

| **Sequence** | **Run 1** | **Run 2** | $\bar{x}$ | **σ** |
|---|---|---|---|---|
| FDY | 17.949 | 17.877 | 17.913 | 0.036 |
| YDF | 17.886 | 17.994 | 17.940 | 0.054 |
| FYD | 17.887 | 17.840 | 17.864 | 0.024 |
| YFD | 16.729 | 18.022 | 17.376 | 0.646 |
| DFY | 16.956 | 17.188 | 17.072 | 0.116 |
| DYF | 16.949 | 17.280 | 17.114 | 0.165 |

Table S4 – Average amidated D/Y/F tripeptide – water hydrogen bonds (sTIP3P, 250 ns)

| **Sequence** | **Run 1** | **Run 2** | $\bar{x}$ | **σ** |
|---|---|---|---|---|
| FDY | 17.421 | 17.575 | 17.498 | 0.077 |
| YDF | 17.729 | 17.578 | 17.654 | 0.075 |
| FYD | 17.858 | 17.825 | 17.842 | 0.017 |
| YFD | 17.135 | 16.971 | 17.053 | 0.082 |
| DFY | 16.589 | 16.570 | 16.580 | 0.009 |
| DYF | 16.591 | 16.857 | 16.724 | 0.133 |

Table S5 – Average amidated D/Y/F tripeptide – water hydrogen bonds (mTIP3P, 500 ns)

| **Sequence** | **Run 1** | **Run 2** | **Run 3** | $\bar{x}$ | **σ** |
|---|---|---|---|---|---|
| FDY | 18.010 | 18.292 | 18.200 | 18.167 | 0.117 |
| YDF | 17.868 | 18.234 | 18.015 | 18.039 | 0.150 |
| FYD | 17.785 | 16.932 | 18.097 | 17.605 | 0.492 |
| YFD | 18.045 | 17.500 | 17.872 | 17.806 | 0.227 |
| DFY | 16.974 | 16.851 | 17.223 | 17.016 | 0.155 |
| DYF | 17.088 | 17.205 | 17.023 | 17.105 | 0.075 |

Table S6 – Average amidated D/Y/F tripeptide – water hydrogen bonds (sTIP3P, 500 ns)

| **Sequence** | **Run 1** | **Run 2** | **Run 3** | $\bar{x}$ | **σ** |
|---|---|---|---|---|---|
| FDY | 17.973 | 17.409 | 17.492 | 17.625 | 0.249 |
| YDF | 17.602 | 17.772 | 17.596 | 17.657 | 0.082 |
| FYD | 17.547 | 16.590 | 17.675 | 17.271 | 0.484 |
| YFD | 16.704 | 17.031 | 17.183 | 16.973 | 0.200 |
| DFY | 16.735 | 16.907 | 16.800 | 16.814 | 0.071 |
| DYF | 16.821 | 16.759 | 16.577 | 16.719 | 0.104 |

Table S7 – Peptide-water model free energy differences ($\Delta G_{HB}$)

| **Sequence** | **$\Delta G_{HB}$ (kJ/mol) 250 ns** | **$\Delta G_{HB}$ (kJ/mol) 500 ns** |
|---|---|---|
| FDY | -0.059 | -0.076 |
| YDF | -0.042 | -0.055 |
| FYD | -0.003 | -0.047 |
| YFD | -0.048 | -0.120 |
| DFY | -0.073 | -0.030 |
| DYF | -0.061 | -0.060 |
| $\bar{x}$ | -0.048 | -0.065 |
| **σ** | 0.022 | 0.028 |

Table S8 – Single molecule ν values for D/Y/F tripeptides in mTIP3P water

| **Sequence** | **Run 1** | **Run 2** | **Run 3** | $\bar{x}$ | **σ** |
|---|---|---|---|---|---|
| FDY | 139 | 211 | 172 | 174 | 25 |
| YDF | 220 | 172 | 197 | 196 | 17 |
| FYD | 387 | 356 | 360 | 368 | 12 |
| YFD | 351 | 362 | 337 | 350 | 9 |
| DFY | 265 | 247 | 271 | 261 | 9 |
| DYF | 266 | 220 | 299 | 262 | 28 |

Table S9 – Single molecule ν values for D/Y/F tripeptides in sTIP3P water

| **Sequence** | **Run 1** | **Run 2** | **Run 3** | $\bar{x}$ | **σ** |
|---|---|---|---|---|---|
| FDY | 129 | 146 | 169 | 148 | 14 |
| YDF | 152 | 160 | 245 | 186 | 36 |
| FYD | 391 | 443 | 400 | 411 | 20 |
| YFD | 441 | 406 | 344 | 397 | 35 |
| DFY | 321 | 295 | 274 | 297 | 17 |
| DYF | 230 | 313 | 190 | 244 | 44 |

Table S10 – Single molecule ν values for FFD in +1, 0, -1 and -2 ionization states in sTIP3P water

| **Sequence** | **Run 1** | **Run 2** | **Run 3** | $\bar{x}$ | **σ** |
|---|---|---|---|---|---|
| FFD (+1) | 326 | 249 | 329 | 301 | 32 |
| FFD (0) | 356 | 382 | 362 | 367 | 10 |
| FFD (-1) | 252 | 269 | 262 | 261 | 6 |
| FFD (-2) | 138 | 129 | 122 | 130 | 6 |

Table S11 – D/Y/F tripeptide aggregation propensities (AP) with mTIP3P water

| Sequence | Run 1 | Run 2 | $\bar{x}$ | σ |
|---|---|---|---|---|
| FDY | 1.597 | 1.512 | 1.554 | 0.042 |
| YDF | 1.625 | 1.573 | 1.599 | 0.026 |
| FYD | 2.124 | 1.998 | 2.061 | 0.063 |
| YFD | 2.061 | 2.004 | 2.032 | 0.028 |
| DFY | 1.924 | 1.733 | 1.828 | 0.095 |
| DYF | 1.855 | 1.781 | 1.818 | 0.037 |
| | | $\bar{x}$ | 1.815 | |
| | | σ | 0.193 | |

Table S12 – D/Y/F tripeptide aggregation propensities (AP) with sTIP3P water

| Sequence | Run 1 | Run 2 | $\bar{x}$ | σ |
|---|---|---|---|---|
| FDY | 2.319 | 2.437 | 2.378 | 0.059 |
| YDF | 2.432 | 2.368 | 2.400 | 0.032 |
| FYD | 2.649 | 2.758 | 2.704 | 0.054 |
| YFD | 2.559 | 2.742 | 2.650 | 0.091 |
| DFY | 2.263 | 2.318 | 2.290 | 0.028 |
| DYF | 2.490 | 2.544 | 2.517 | 0.027 |
| | | $\bar{x}$ | 2.490 | |
| | | σ | 0.149 | |

Table S13 – Histogram derived single molecule ν values for D/Y/F tripeptides in mTIP3P water

| Sequence | Run 1 | Run 2 | Run 3 | $\bar{x}$ | σ |
|---|---|---|---|---|---|
| FDY | 89 | 173 | 129 | 130 | 30 |
| YDF | 154 | 139 | 125 | 139 | 10 |
| FYD | 277 | 285 | 281 | 281 | 3 |
| YFD | 228 | 265 | 270 | 254 | 16 |
| DFY | 177 | 176 | 208 | 187 | 13 |
| DYF | 184 | 185 | 197 | 189 | 5 |

Table S14 – Histogram derived single molecule ν values for D/Y/F tripeptides in sTIP3P water

| Sequence | Run 1 | Run 2 | Run 3 | $\bar{x}$ | σ |
|---|---|---|---|---|---|
| FDY | 92 | 97 | 127 | 105 | 13 |
| YDF | 98 | 114 | 155 | 122 | 21 |
| FYD | 289 | 332 | 265 | 295 | 24 |
| YFD | 319 | 293 | 251 | 288 | 24 |
| DFY | 216 | 212 | 174 | 201 | 16 |
| DYF | 187 | 240 | 141 | 189 | 35 |

Table S15 – Single molecule ν values for FFD in +1, 0, -1 and -2 ionization states in sTIP3P water using NVT ensemble

| Sequence | Run 1 | Run 2 | Run 3 | $\overline{x}$ | σ |
|---|---|---|---|---|---|
| FFD (+1) | 255 | 290 | 302 | 282 | 17 |
| FFD (0) | 375 | 387 | 331 | 364 | 21 |
| FFD (-1) | 232 | 291 | 282 | 268 | 22 |
| FFD (-2) | 130 | 132 | 122 | 128 | 4 |

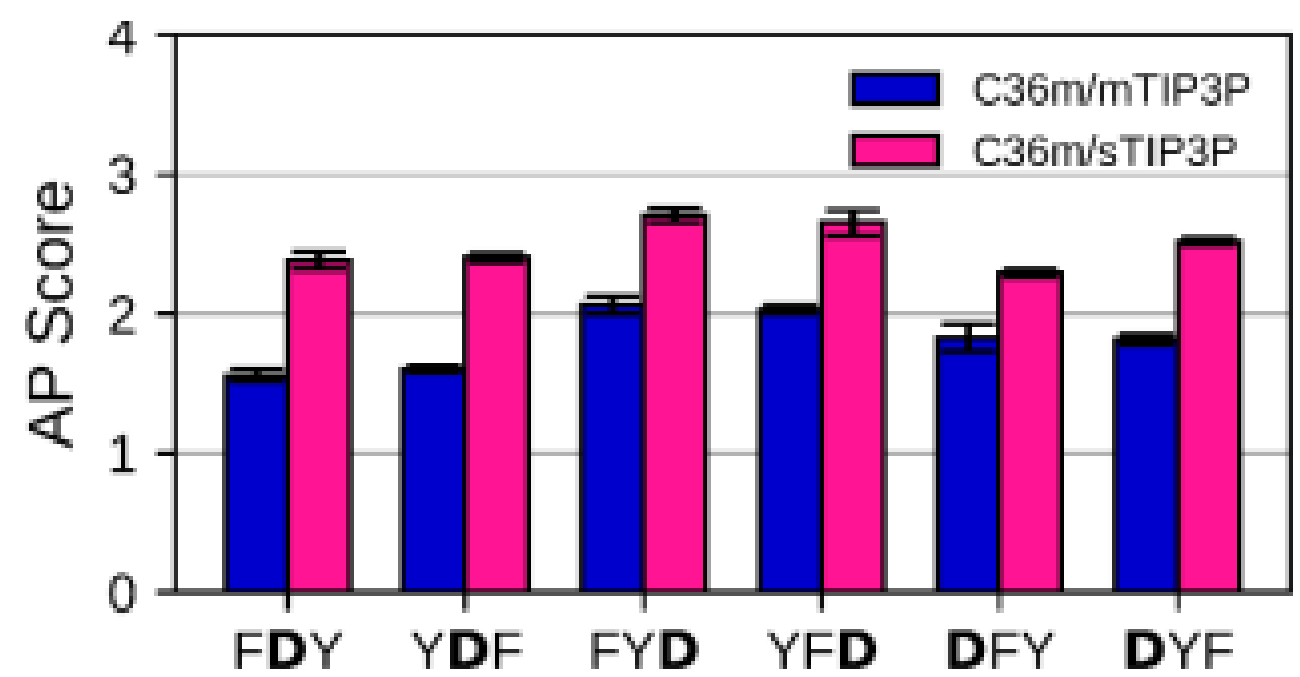


**Figure S1.** Aggregation propensity (AP) scores for multi-molecule simulations (70 peptides) of the amidated D/Y/F tripeptides (Table S11, S12) in mTIP3P or sTIP3P water using the Charmm36m (C36m) forcefield. Mean and standard deviations are calculated from duplicate simulations. The mTIP3P water model leads to a systematic reduction in average AP score compared to the sTIP3P model.

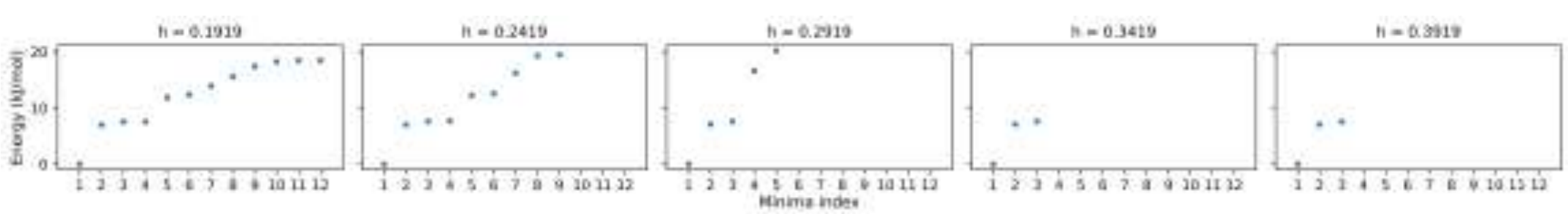


**Figure S2.** Illustration of the number of minima, sorted with respect to energy, within a FEL compared to the bandwidth value used. A small bandwidth (h = 0.1919) results in a rough FEL with many local minima, conversely a large bandwidth (h = 0.3919) results in a smooth FEL

with few minima (Figure S3). In this study we selected the intermediate value 0.291935 which has an intermediate number of minima.

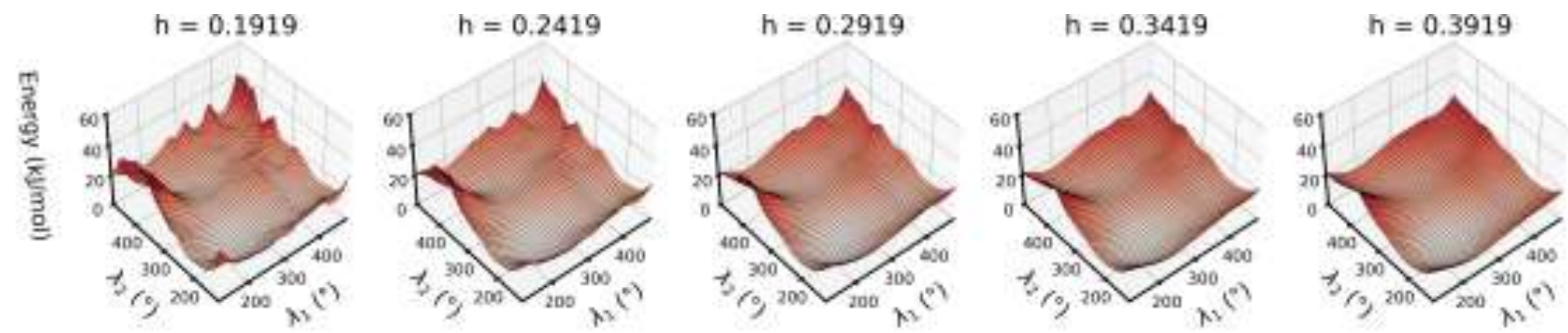


**Figure S3.** Influence of bandwidth on FEL smoothness illustrating again the need to choose an intermediate value between a very rough (h = 0.1919) and very smooth (h = 0.3919) value.

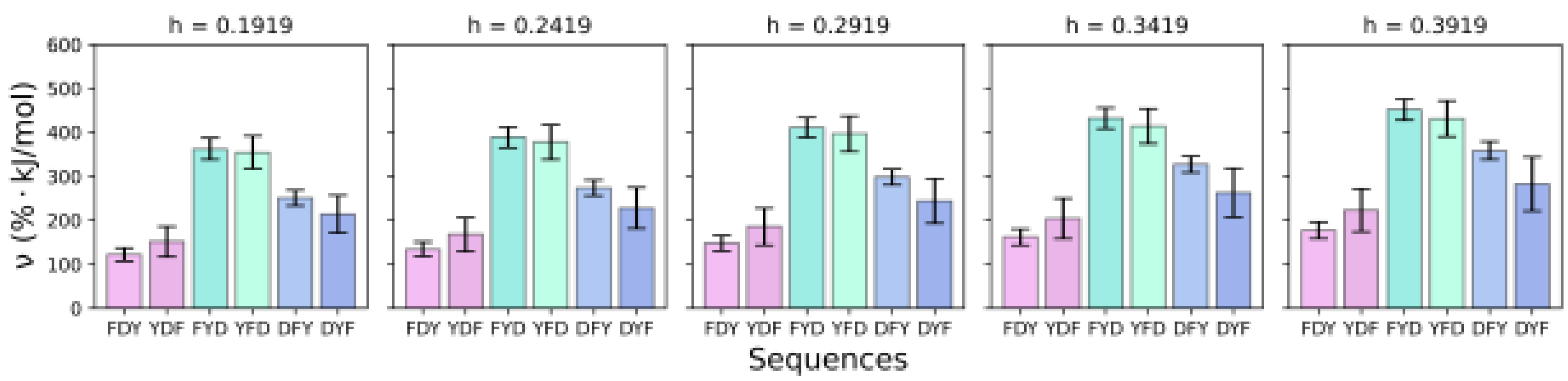


**Figure S4.** Relative influence of bandwidth on measured $\nu$ value for the amidated D/Y/F tripeptides with the sTIP3P water model. This shows that as the smoothness of the underlying FEL increases so too does the measured $\nu$ value and that the relative ordering of the sequences does not change with respect to the selected bandwidth parameter.

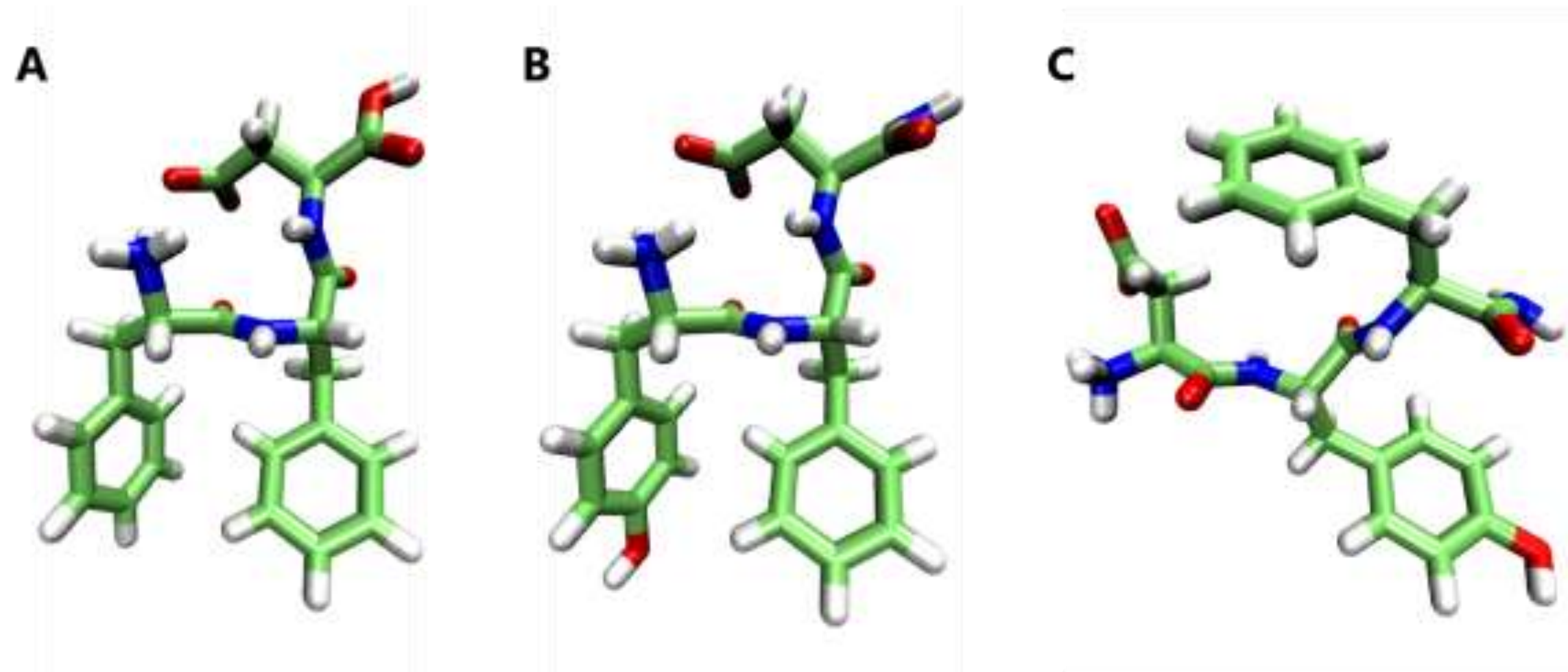


**Figure S5.** Crystal structure conformations of (A) FFD, (B) YFD and (C) DYF. Illustrating the strong structural similarity between FFD and YFD. Note that the unit cell of both YFD and DYF contains two unique conformers however these are very structurally similar (see proximity in $\lambda_1/\lambda_2$ plot, Figure 2B) and only one is included here for simplicity (CCDC deposition numbers: 1539905 and 1539906 for DYF and YFD respectively).

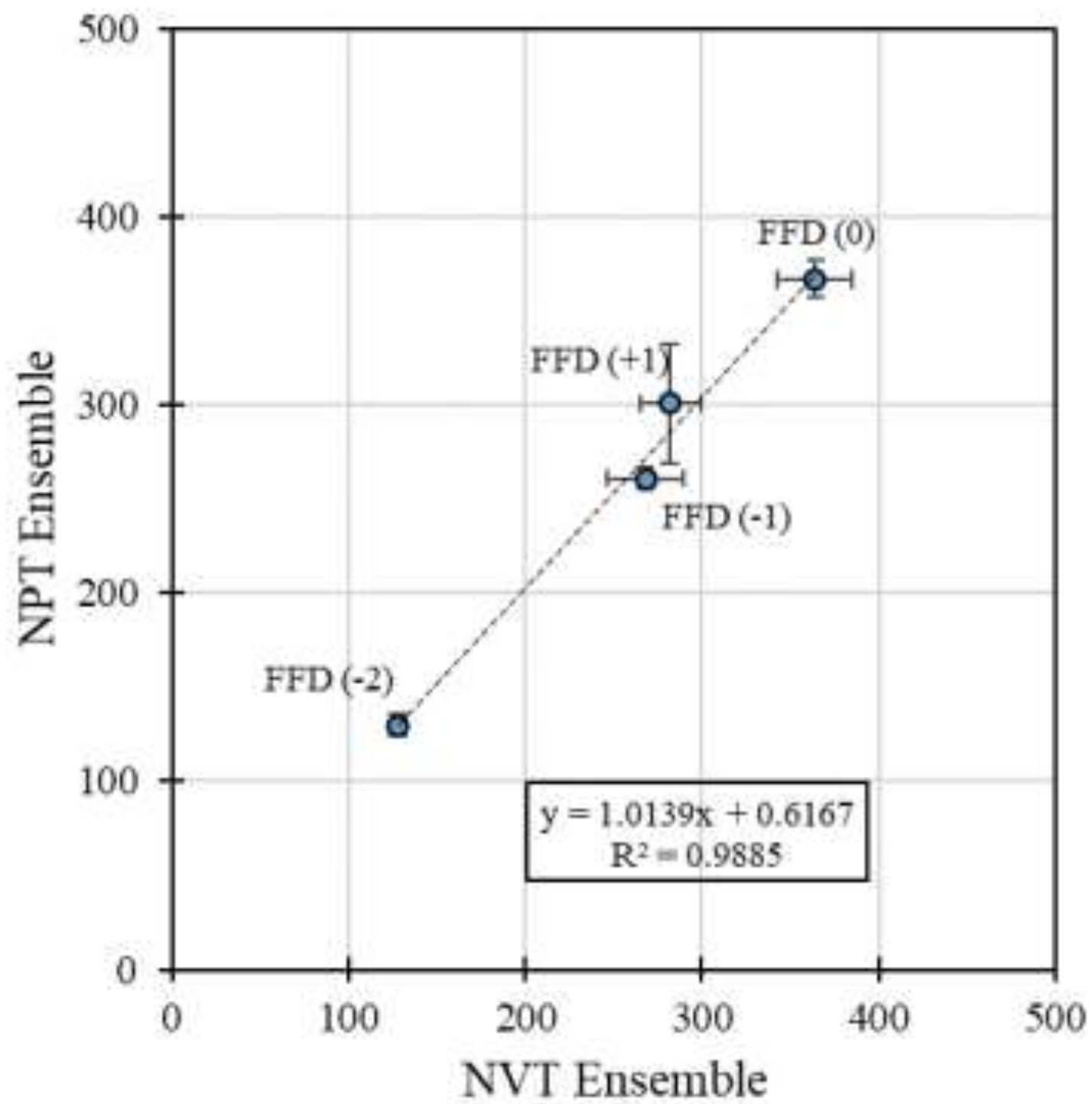


**Figure S6.** Comparison of ν values obtained for FFD in +1, 0, -1 and -2 ionization states in sTIP3P water using canonical (NVT) or isothermal-isobaric (NPT) ensembles (Table S10, S15).

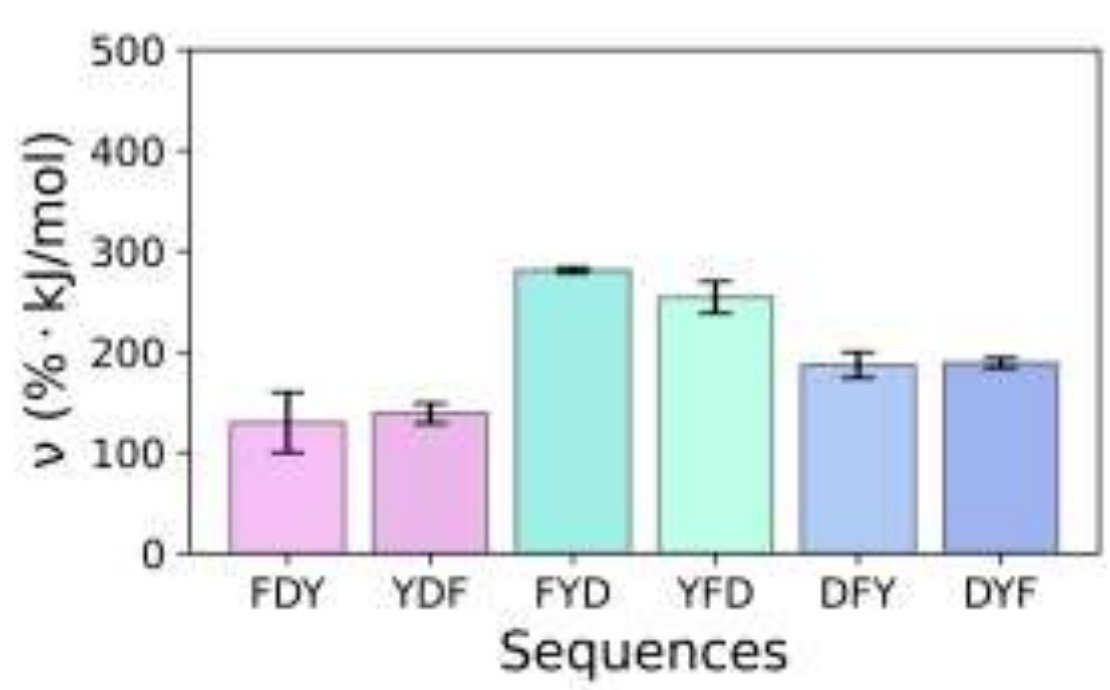


**Figure S7.** Histogram derived single molecule ν values for D/Y/F tripeptides in mTIP3P water (Table S13).

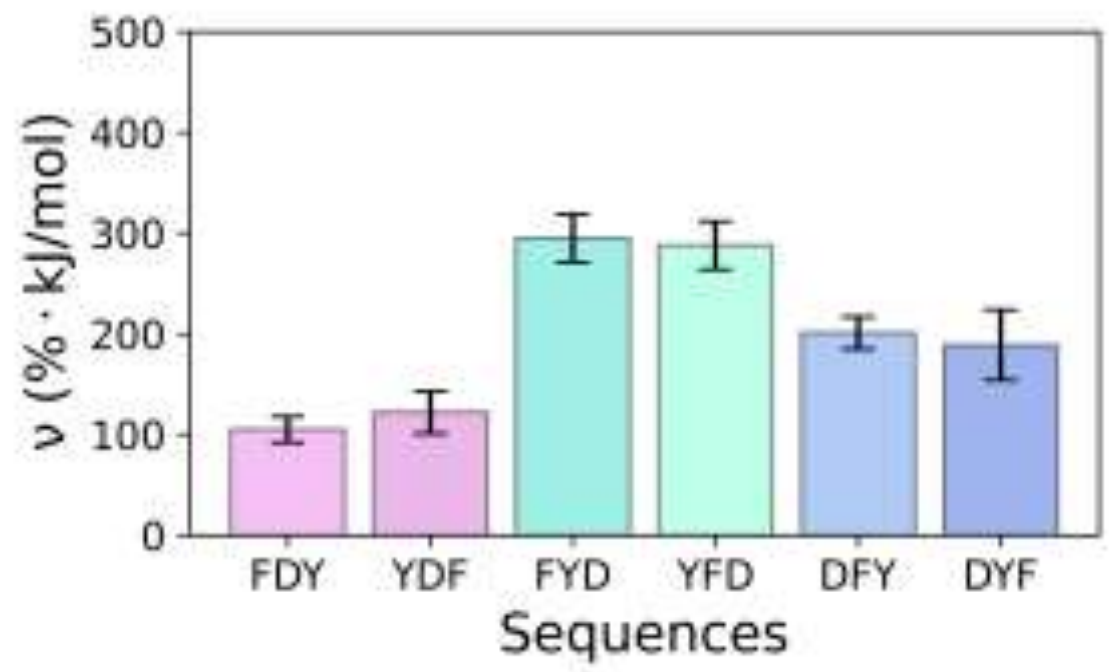


**Figure S8.** Histogram derived single molecule ν values for D/Y/F tripeptides in sTIP3P water (Table S14).

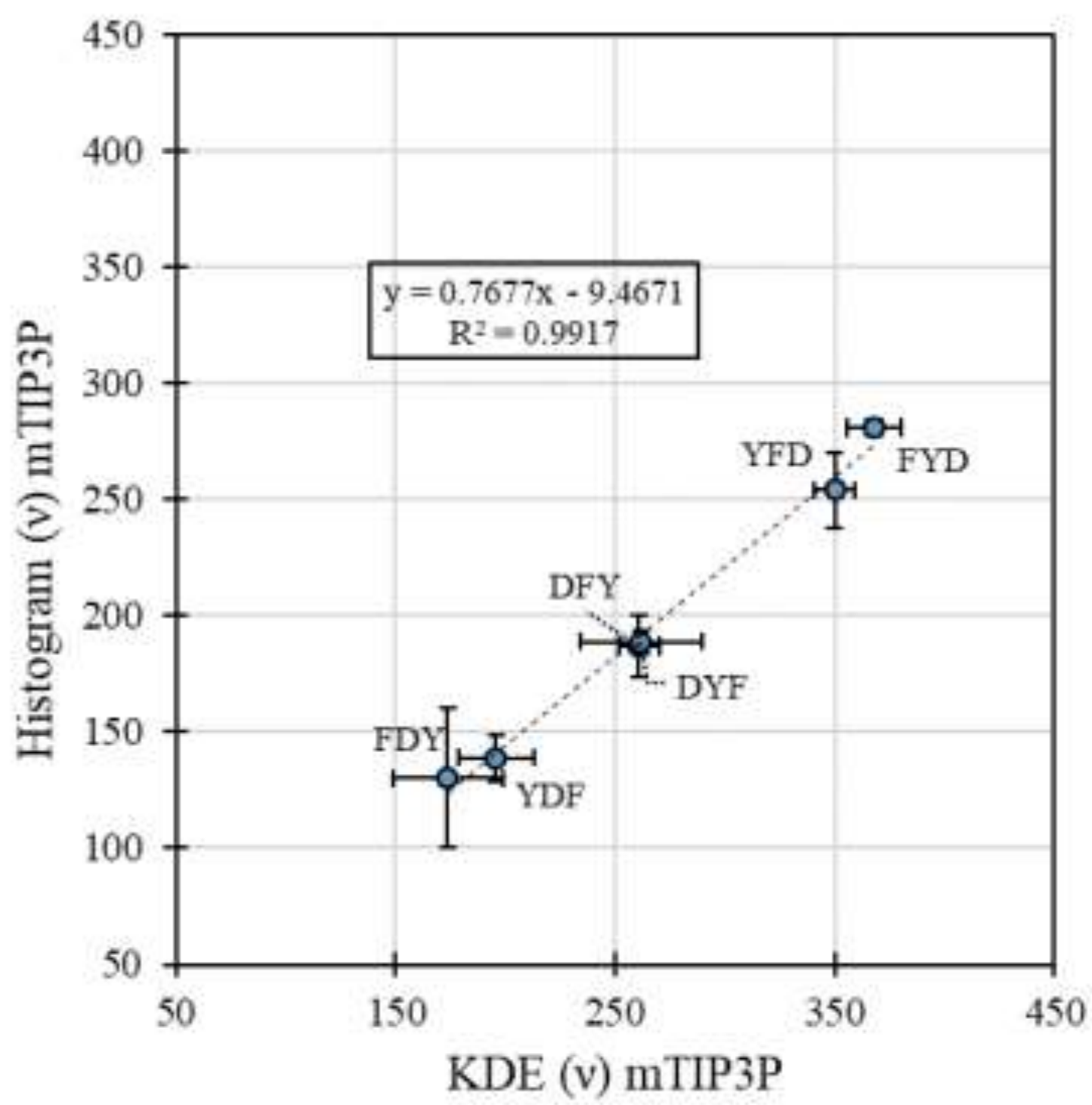


**Figure S9.** Comparison of ν values obtained using free energy landscapes constructed using kernel density estimator (KDE) or histogram binning, for D/Y/F tripeptides simulated using the mTIP3P water model (Table S8, S13).

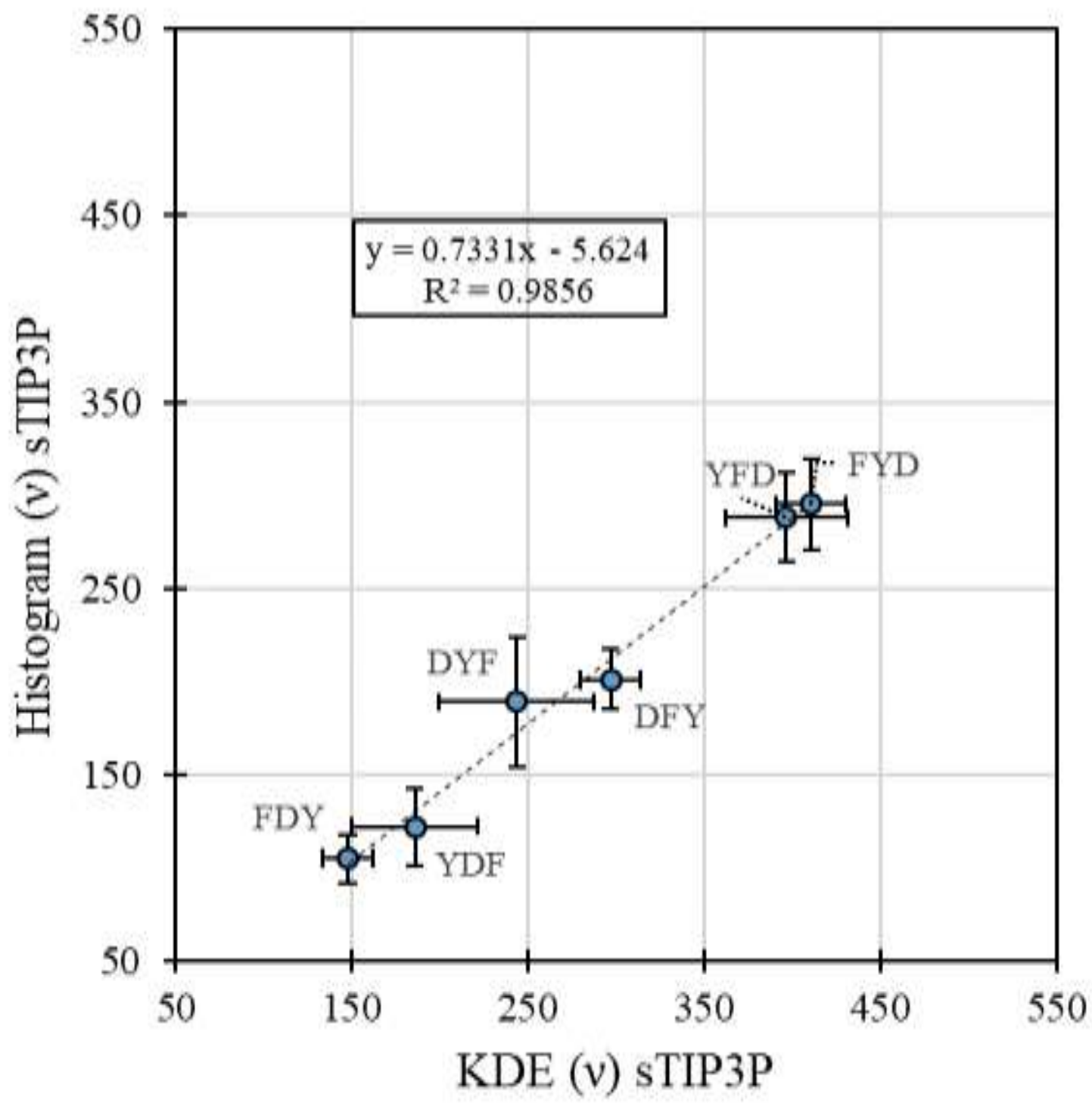


**Figure S10.** Comparison of ν values obtained using free energy landscapes constructed using kernel density estimator (KDE) or histogram binning, for D/Y/F tripeptides simulated using the sTIP3P water model (Table S9, S14).

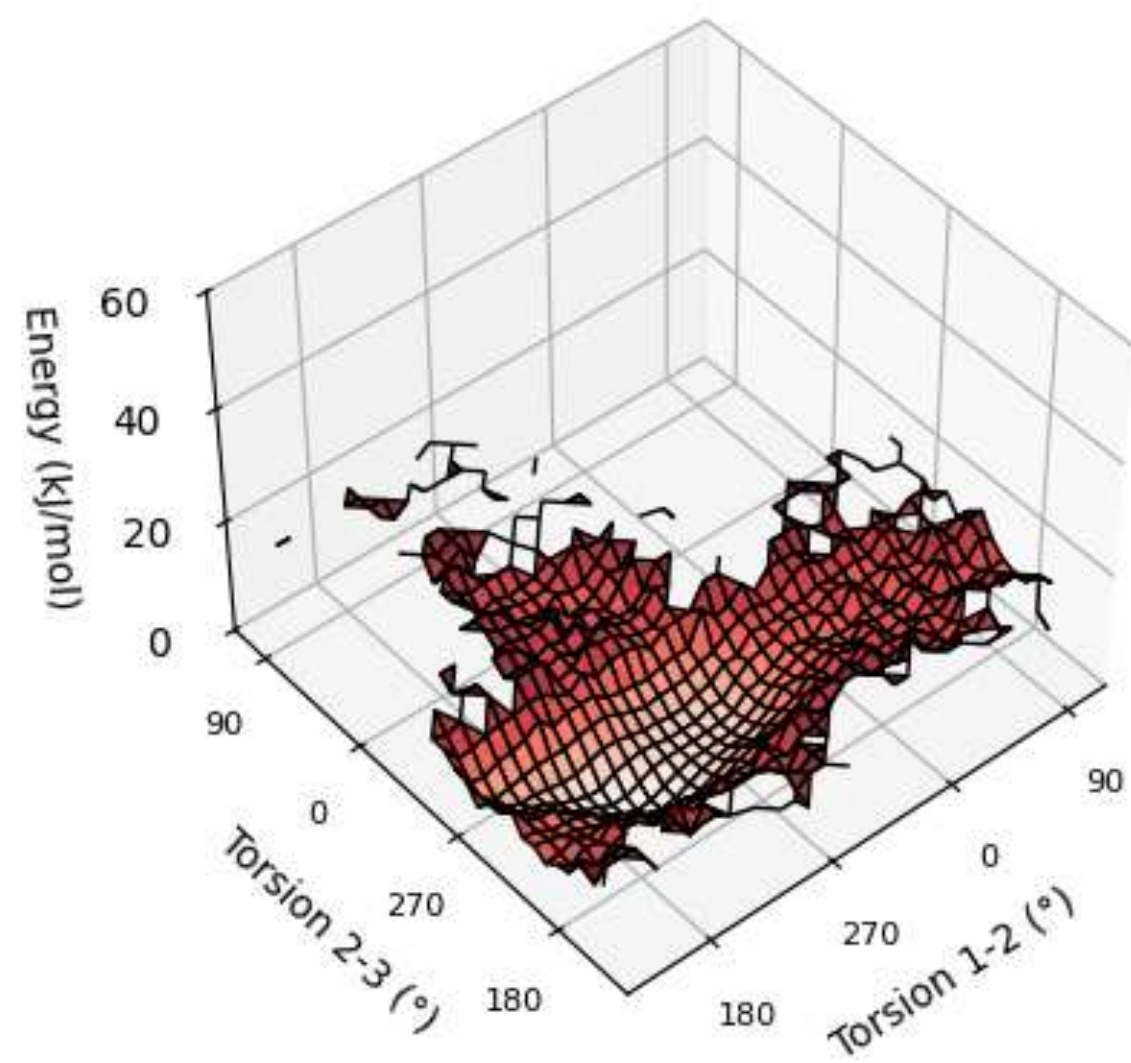


**Figure S11.** Free energy landscape obtained by histogramming for FDY showing higher degree of roughness compared to those obtained using the KDE method, bin range [120°, 480°] and spacing of 10° (*i.e.*, 36 x 36 bins total).

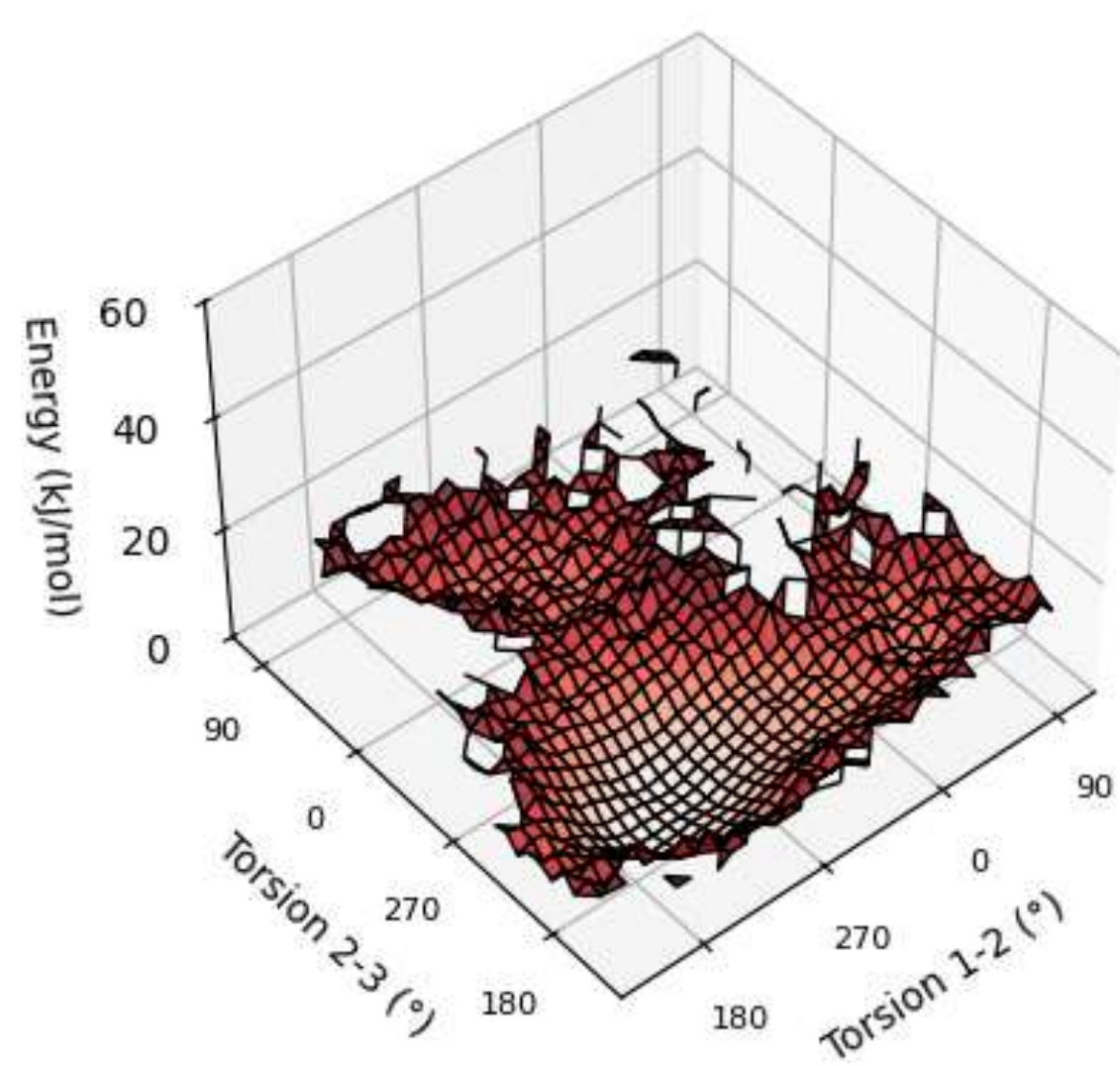

**Figure S12.** Free energy landscape obtained by histogramming for DYF showing higher degree of roughness compared to those obtained using the KDE method, bin range [120°, 480°] and spacing of 10° (*i.e.*, 36 x 36 bins total).

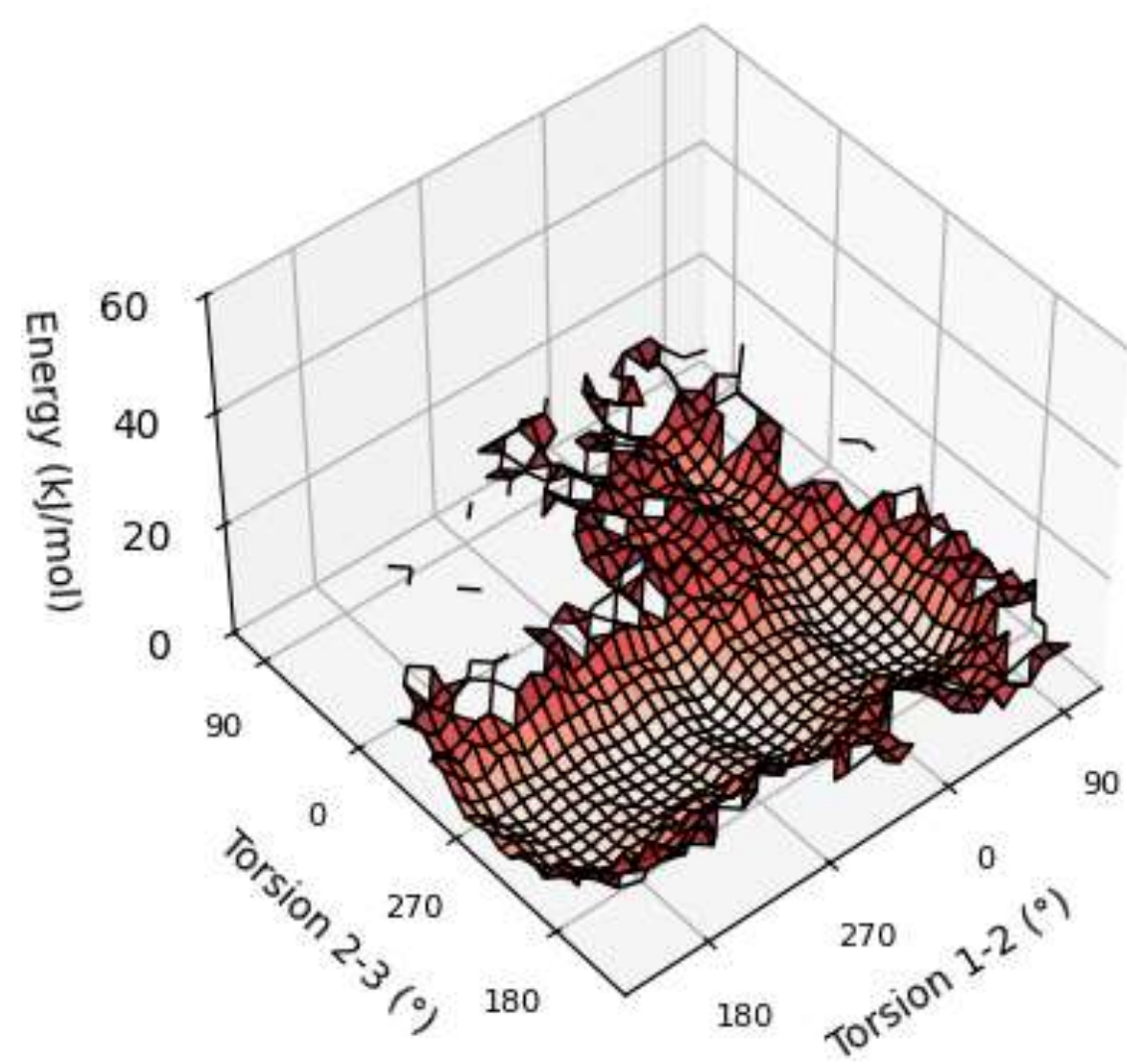


**Figure S13.** Free energy landscape obtained by histogramming for YFD showing higher degree of roughness compared to those obtained using the KDE method, bin range [120°, 480°] and spacing of 10° (*i.e.*, 36 x 36 bins total).
